# Performing different kinds of physical exercise differentially attenuates the genetic effects on obesity measures: Evidence from 18,424 Taiwan Biobank participants

**DOI:** 10.1371/journal.pgen.1008277

**Published:** 2019-08-01

**Authors:** Wan-Yu Lin, Chang-Chuan Chan, Yu-Li Liu, Albert C. Yang, Shih-Jen Tsai, Po-Hsiu Kuo

**Affiliations:** 1 Institute of Epidemiology and Preventive Medicine, College of Public Health, National Taiwan University, Taipei, Taiwan; 2 Department of Public Health, College of Public Health, National Taiwan University, Taipei, Taiwan; 3 Institute of Occupational Medicine and Industrial Hygiene, College of Public Health, National Taiwan University, Taipei, Taiwan; 4 Center for Neuropsychiatric Research, National Health Research Institutes, Zhunan, Miaoli County, Taiwan; 5 Division of Psychiatry, National Yang-Ming University, Taipei, Taiwan; 6 Division of Interdisciplinary Medicine and Biotechnology, Beth Israel Deaconess Medical Center/Harvard Medical School, Boston, Massachusetts, United States of America; 7 Institute of Brain Science, National Yang-Ming University, Taipei, Taiwan; 8 Department of Psychiatry, Taipei Veterans General Hospital, Beitou District, Taipei, Taiwan; University of Copenhagen, DENMARK

## Abstract

Obesity is a worldwide health problem that is closely linked to many metabolic disorders. Regular physical exercise has been found to attenuate the genetic predisposition to obesity. However, it remains unknown what kinds of exercise can modify the genetic risk of obesity. This study included 18,424 unrelated Han Chinese adults aged 30–70 years who participated in the Taiwan Biobank (TWB). A total of 5 obesity measures were investigated here, including body mass index (BMI), body fat percentage (BFP), waist circumference (WC), hip circumference (HC), and waist-to-hip ratio (WHR). Because there have been no large genome-wide association studies on obesity for Han Chinese, we used the TWB internal weights to construct genetic risk scores (GRSs) for each obesity measure, and then test the significance of GRS-by-exercise interactions. The significance level throughout this work was set at 0.05/550 = 9.1x10^-5^ because a total of 550 tests were performed. Performing regular exercise was found to attenuate the genetic effects on 4 obesity measures, including BMI, BFP, WC, and HC. Among the 18 kinds of self-reported regular exercise, 6 mitigated the genetic effects on at least one obesity measure. Regular jogging blunted the genetic effects on BMI, BFP, and HC. Mountain climbing, walking, exercise walking, international standard dancing, and a longer practice of yoga also attenuated the genetic effects on BMI. Exercises such as cycling, stretching exercise, swimming, dance dance revolution, and qigong were not found to modify the genetic effects on any obesity measure. Across all 5 obesity measures, regular jogging consistently presented the most significant interactions with GRSs. Our findings show that the genetic effects on obesity measures can be decreased to various extents by performing different kinds of exercise. The benefits of regular physical exercise are more impactful in subjects who are more predisposed to obesity.

## Author summary

The complex interplay of genetics and lifestyle makes obesity a challenging issue. Previous studies have found performing regular physical exercise could blunt the genetic effects on body mass index (BMI). However, BMI does not take into account lean body mass or identify central obesity. Moreover, it remains unclear what kinds of exercise could more effectively attenuate the genetic effects on obesity measures. With a sample of 18,424 unrelated Han Chinese adults, we comprehensively investigated gene-exercise interactions on 5 obesity measures: BMI, body fat percentage, waist circumference, hip circumference, and waist-to-hip ratio. Moreover, we tested whether the genetic effects on obesity measures could be modified by any of 18 kinds of self-reported regular exercise. Because no large genome-wide association studies on obesity have been done for Han Chinese, we constructed genetic risk scores with internal weights for analyses. Among these exercises, regular jogging consistently presented the strongest evidence to mitigate the genetic effects on all 5 obesity measures. Moreover, mountain climbing, walking, exercise walking, international standard dancing, and a longer practice of yoga attenuated the genetic effects on BMI. The benefits of regularly performing these 6 kinds of exercise are more impactful in subjects who are more predisposed to obesity.

## Introduction

Obesity is one of the most challenging public health issues worldwide [1–6]. According to the World Health Organization, a person with a body mass index (BMI) of 30 kg/m^2^ or above is generally considered obese. Although BMI is easy to calculate and is commonly used to identify obesity, it does not take into account lean body mass or identify central obesity. Four important metrics, body fat percentage (BFP), waist circumference (WC), hip circumference (HC), and waist-to-hip ratio (WHR), are complementary to BMI. The BFP of an individual is the total fat mass divided by the total body mass, multiplied by 100. HC is a useful predictor of metabolic syndromes such as diabetes [7]. WC and WHR are indicators of central obesity [8].

Obesity is complicated as it is caused by genetics, lifestyle, and the interplay between them [9, 10]. The heritability of BMI was reported to range from 24% to 81% [11], and many genes have been shown to be related to obesity [12]. Although hereditary factors are critical, some lifestyle factors can modify the genetic influences on BMI [13–24]. For example, regular physical exercise has been found to blunt the genetic effects on obesity [13–16, 18, 20, 24]. However, most of these studies focused on only BMI, without discussing central obesity. Moreover, investigations specific to particular kinds of exercise remain limited. It is unknown what kinds of exercise (jogging, mountain climbing, cycling, etc.) can attenuate the genetic effects on obesity measures. To fill the research gap, we here comprehensively investigated gene-exercise interactions on the 5 obesity measures: BMI, BFP, WC, HC, and WHR. Moreover, we investigated whether 18 kinds of exercise could modify the associations between genetic risk scores (GRSs) and these 5 obesity measures.

## Materials and methods

### Ethics statement

TWB received ethical approval from the Institutional Review Board on Biomedical Science Research/IRB-BM, Academia Sinica, Taiwan, and from the Ethics and Governance Council of Taiwan Biobank, Taiwan. Written informed consent was obtained from each participant in accordance with institutional requirements and the principles of the Declaration of Helsinki. Moreover, the current study was approved by the Research Ethics Committee of National Taiwan University Hospital (NTUH-REC no. 201805050RINB).

### Taiwan Biobank

Taiwan Biobank (TWB) is the largest government-supported biobank in Taiwan. The aim of TWB is to collect lifestyle and genomic data from Taiwan residents [25, 26]. TWB keeps recruiting community-based volunteers who are 30 to 70 years of age and have no history of cancers. Participants signed informed consent, provided blood samples and a range of information via a face-to-face interview and physical examination. Our study comprised 20,287 TWB individuals who have been whole-genome genotyped until October, 2018. To remove cryptic relatedness, we estimated the genome-wide identity by descent (IBD) sharing coefficients between any two subjects. The IBD scores for all pairs of subjects, i.e., PI-HAT = Probability(IBD = 2) + 0.5×Probability(IBD = 1), were obtained from PLINK 1.9 [27]. Similar to many genetic studies [28–30], we excluded third-degree relatives by removing one individual from a pair with PI-HAT ≥ 0.125. After this step, 18,424 unrelated subjects (9,093 males and 9,331 females) remained in our analysis.

The majority of TWB subjects were of Han Chinese ancestry [25]. The TWB chip is based on Axiom Genome-Wide Array Plate System (Affymetrix, Santa Clara, CA, USA). It genotyped a total of 646,783 autosomal single-nucleotide polymorphisms (SNPs). We excluded 51,293 SNPs with genotyping rates < 95%, 6,095 SNPs with Hardy-Weinberg test *P*-values < 5.7×10^−7^ [31], and 1,869 variants with minor allele frequencies (MAFs) < 1%. The remaining 587,526 SNPs were used to construct ancestry principal components (PCs) for the adjustment of population stratification.

The TWB measured body height and weight for each participant. BMI was calculated by weight (kg)/[height (m)]^2^. In addition to BMI, 4 measures including BFP, WC, HC, and WHR were also investigated. BFP is the percentage of an individual’s weight that is made up of fat. WHR is the ratio of WC to HC and is a commonly used index for central obesity [8].

In addition to a physical examination, each participant completed a questionnaire through a face-to-face interview with one of the TWB researchers. Questions addressed personal information and lifestyle factors. Regular exercise was defined as engaging in 30 minutes of “exercise” three times a week. “Exercise” included only leisure-time activities such as jogging, yoga, mountain climbing, cycling, swimming, dance dance revolution (DDR, a computer game based on dancing with music videos), playing basketball, etc. Occupational activities such as physical work or heavy manual work were not counted as “exercise”.

### Covariates adjusted in all models

Sex and age (in years) have been considered as important covariates in most obesity studies [13–16, 18, 20, 24, 32–34]. Moreover, some studies also adjusted for drinking status, smoking status, and educational attainment [16]. A previous large-scale study has found an inverse association between BMI as well as WC and education level [35]. Therefore, we also considered educational attainment as one of the covariates for obesity measures. Educational attainment was recorded as a value ranging from 1 to 7, where 1 indicated “illiterate”, 2 meant “no formal education but literate”, 3 represented “primary school graduate”, 4 indicated “junior high school graduate”, 5 meant “senior high school graduate”, 6 represented “college graduate”, and 7 indicated “Master’s or higher degree”.

Drinking was defined as a subject having a weekly intake of more than 150 cc of alcohol for at least 6 months and having not stopped drinking at the time his/her obesity measures were being assessed. Smoking was defined as a subject who had smoked for at least 6 months and had not quit smoking at the time his/her obesity measures were being assessed.

### Genetic risk scores (GRS) for the five obesity measures

In most gene-environment interaction (G×E) studies, investigators typically constructed a GRS and tested the significance of the GRS×E interaction term (E represents the environmental factor) [13–24]. A GRS was a weighted sum of risk-allele counts, where the weights were usually retrieved from large published genome-wide association studies (GWASs) or meta-analyses [13–24]. Recent G×E studies related to obesity measures [14, 16–19, 21, 23] usually constructed a GRS according to the results of a large meta-analysis [34], in which 97 BMI-associated SNPs reaching the genome-wide significance level (*p* < 5×10^−8^) were reported [34].

A total of 20 out of the 97 SNPs were genotyped in the TWB chip. We imputed the genotypes of other SNPs using the Michigan Imputation Server (https://imputationserver.sph.umich.edu/index.html), with the reference panel based on the East Asian (EAS) population from the 1000 Genomes Phase 3 v5. After removing SNPs with MAFs < 1% and SNPs with Hardy-Weinberg test *P*-values < 5.7×10^−7^ [31], 86 SNPs remained in S1 Table. The European-based GRS was calculated as $EuGRS=\sum_{j=1}^{86}w_{j}{SNP}_{j}$, where the weights (*w*_*j*_,*j* = 1,⋯,86) were the effect sizes reported by Locke *et al*. [34], and *SNP*_*j*_ was the number of effect alleles at the *j*^th^ SNP. Each EuGRS was then transformed into a *z*-score that indicated how many standard deviations an EuGRS was from the mean. Although EuGRS is positively associated with the 5 obesity measures (S2 Table) (the results of EuGRS×exercise interactions can be found from S3–S5 Tables), it may not be an efficient GRS to detect TWB G×E for the following three reasons.

First, the 97 SNPs account for 2.70% of BMI variation in Europeans [34]. However, in TWB subjects, these SNPs can only explain 1.92%, 1.05%, 1.43%, 1.60%, and 0.79% of variation of BMI, BFP, WC, HC, and WHR, respectively (S6 Table). Second, all the 97 BMI-associated SNPs reached the genome-wide significance level (*p* < 5×10^−8^) in Europeans. However, in TWB, only *rs1558902* located in the fat mass and obesity-associated (*FTO*) gene was associated with BMI at the genome-wide significance level, and only 29 were associated with BMI at the significance level of 0.05 (S1 Table). Third, none of the 97 BMI-associated SNPs were associated with the other 4 obesity measures at the genome-wide significance level (S1 Table). BMI is the most commonly investigated obesity measure. SNPs robustly associated with other obesity measures have not been reported.

Based on the above three reasons, using EuGRS may be inefficient for Han Chinese and for obesity measures other than BMI. However, large obesity-related GWASs in Han Chinese are unavailable. To overcome this problem, we used internal weights to construct a GRS, and then tested the GRS×E interaction term in a regression model. This approach has been proposed in genome-wide [36], pathway-based [37, 38], and gene-based G×E studies [39, 40].

Initially, SNPs in high linkage disequilibrium (LD) were first pruned to avoid multicollinearity [41, 42]. We used PLINK 1.9 command “plink--bfile TWBGWAS--chr 1–22--indep 50 5 2” to prune SNPs in high LD [27]. In this way, we removed SNPs with a variance inflation factor > 2 within a sliding window of size 50, where the sliding window was shifted at each step of 5 SNPs. After this pruning stage, 142,040 SNPs remained. We then regressed BMI on each of the 142,040 SNPs while adjusting for covariates including sex, age, educational attainment, drinking status, smoking status, and the first 10 PCs. The 142,040 regression models were built as follows:

$$BMI=\beta_{0}+\beta_{SNP,i}{SNP}_{i}+\beta_{C}Covariates+\varepsilon ,\;i=1,\cdots ,142040,$$ (1)

where *SNP*_*i*_ is the number of minor alleles at the *i*^th^ SNP (0, 1, or 2) and *ε* is the error term. By testing *H*_0_: *β*_*SNP*,*i*_ = 0 *vs*. *H*_1_: *β*_*SNP*,*i*_ ≠ 0, we obtained a *P*-value regarding the marginal association of the *i*^th^ SNP with BMI.

Considering the model incorporating SNP-by-environment interactions, as follows:

$$BMI=\gamma_{0}+\gamma_{SNP,i}{SNP}_{i}+\gamma_{E}E+\gamma_{Int,i}{SNP}_{i}\times E+\gamma_{C}Covariates+\varepsilon ,\;i=1,\cdots ,142040,$$ (2)

${\hat{\beta }}_{SNP,i}$ (estimated from model 1) and ${\hat{\gamma }}_{Int,i}$ (estimated from model 2) are asymptotically independent under the null hypothesis of no SNP-by-environment interaction (proved in corollary 1 of [43]). A two-stage approach that first filters SNPs by a criterion independent of the test statistic (${\hat{\gamma }}_{Int,i}$ estimated from model 2) under the null hypothesis, and then only uses SNPs that pass the filter, can maintain type I error rates and boost power [44, 45].

Given a *P*-value threshold (a filter), the 142,040 SNPs were allocated into a BMI-associated set and a BMI-unassociated set according to their marginal-association *P*-values. Suppose there were *m* SNPs associated with BMI, the BMI genetic risk score (BMIGRS) was calculated as $\sum_{j=1}^{m}{\hat{\beta }}_{SNP,j}{SNP}_{j}$, where the weights (${\hat{\beta }}_{SNP,j},j=1,\cdots ,m$) had been estimated from model (1), and *SNP*_*j*_ was the number of minor alleles at the *j*^th^ SNP in the BMI-associated set.

Because BMI-unassociated SNPs were filtered out from the construction of BMIGRS, this approach is the so-called “marginal-association filtering” in G×E analyses [40, 43, 45]. Following the suggestion from our previous methodological study [36], 10 *P*-value thresholds were considered: 0.0001, 0.00025, 0.0005, 0.001, 0.0025, 0.005, 0.01, 0.025, 0.05, and 0.1. S7 Table shows the numbers of SNPs in the BMI-associated sets under the 10 *P*-value thresholds. For each TWB subject, 10 BMIGRSs were calculated based on the 10 sets of SNPs. For example, the 9^th^ BMIGRS accumulated the information of 7,753 SNPs (S7 Table).

Similar with model (1), BFP, WC, HC and WHR were regressed on each of the 142,040 SNPs while adjusting for the same covariates, respectively. A total of 10 BFPGRSs, 10 WCGRSs, 10 HCGRSs, and 10 WHRGRSs were obtained under the 10 *P*-value thresholds. Each GRS was then transformed into a *z*-score that indicated how many standard deviations a GRS was from the mean. The number of SNPs to form each GRS was listed in S7 Table.

### The GRS approach based on marginal effects of SNPs (GRS-M)

We investigated whether the association of BMIGRS with BMI could be modified by regular physical exercise (yes or no). BMI was regressed on a BMIGRS, regular exercise or not (E: 1 vs. 0), and the interaction between them (BMIGRS×E), while adjusting for sex, age, educational attainment, drinking status, smoking status, and the first 10 PCs. The regression model was built as follows:

$$BMI=\beta_{0}+\beta_{GRS}BMIGRS+\beta_{E}E+\beta_{Int}BMIGRS\times E+\beta_{C}Covariates+\varepsilon .$$ (3)

With 10 BMIGRSs, 10 regression models like (3) were fitted and 10 *P*-values regarding testing *H*_0_: *β*_*Int*_ = 0 *vs*. *H*_1_: *β*_*Int*_ ≠ 0 were obtained. To adjust for multiple testing, the Bonferroni-corrected *P*-value was calculated as 10 times the minimum *P*-value of the 10 BMIGRS×E interaction tests. This approach is called “the GRS approach based on marginal effects of SNPs”, abbreviated as the “GRS-M” method [36]. The comprehensive simulations performed by Hüls *et al*. [37, 38] and Lin *et al*. [36] have confirmed the validity of building GRS with marginal effects of SNPs in detecting G×E. Extracting weights from other cohorts or splitting data in two subsets is not required for the GRS-M approach [36]. The GRS-M approach is valid in the sense that the empirical type I error rate is satisfactorily controlled. Furthermore, it is generally the most powerful test if some phenotype-associated SNPs also exhibit interactions with E [36].

Similarly, we also investigated GRS-exercise interactions on the other 4 obesity measures. The significance level throughout this work was set at 0.05/550 = 9.1x10^-5^ because 275 tests for GRS-exercise interactions and 275 tests for main effects of exercises were performed.

## Results

### Basic characteristics of the TWB subjects

Table 1 presents the basic characteristics of the TWB subjects, stratified by the quartiles of the 9^th^ BMIGRS. The aim of this study was to test whether the genetic effects on obesity measures can be modified by any of 18 kinds of exercise. A previous large-scale study has found an inverse association between BMI as well as WC and education level [35]. Our TWB analysis results also show improvements when including educational attainment as a covariate for all 5 obesity measures. By including educational attainment as a covariate, the adjusted R-square increased from 5.9% to 7.3% for BMI, from 34.8% to 35.9% for BFP, from 14.3% to 15.6% for WC, from 4.5% to 4.8% for HC, and from 23.2% to 24.6% for WHR, respectively.

**Table 1:** Basic characteristics stratified by the quartiles of the 9^th^ BMIGRS (marginal-association *P*-value threshold = 0.05).

	Overall	Q1 (lower BMIGRS)	Q2	Q3	Q4 (higher BMIGRS)
**Total, *n***	18 424	4 606	4 606	4 606	4 606
**Males, *n* (%)**	9 093 (49.4)	2 196 (47.7)	2 246 (48.8)	2 381 (51.7)	2 270 (49.3)
**Age (years), mean (s.d.)**	48.9 (11.0)	48.0 (11.3)	49.3 (10.9)	49.4 (10.9)	48.9 (10.8)
**Educational attainment (s.d.)**	5.46 (0.99)	5.45 (0.97)	5.46 (1.00)	5.47 (0.99)	5.44 (1.00)
**Drinking, *n* (%)**	1 345 (7.3)	316 (6.9)	323 (7.0)	358 (7.8)	348 (7.6)
**Smoking, *n* (%)**	2 134 (11.6)	528 (11.5)	541 (11.7)	525 (11.4)	540 (11.7)
**Regular exercise, *n* (%)**	7 652 (41.5)	1 900 (41.3)	2 023 (43.9)	1 922 (41.7)	1 807 (39.2)
**BMI (kg m^-2^) (s.d.)**	24.31 (3.66)	21.22 (2.20)	23.10 (2.19)	24.77 (2.33)	28.15 (3.53)
**Body fat % (s.d.)**	27.29 (7.38)	22.63 (5.88)	25.68 (5.71)	27.98 (6.11)	32.89 (7.57)
**Waist circumference (cm) (s.d.)**	83.93 (10.03)	76.88 (7.50)	81.32 (7.66)	85.09 (7.89)	92.43 (9.74)
**Hip circumference (cm) (s.d.)**	96.34 (6.90)	91.49 (4.84)	94.39 (4.93)	97.11 (5.17)	102.40 (7.16)
**Waist-to-hip ratio (s.d.)**	0.87 (0.068)	0.84 (0.063)	0.86 (0.063)	0.88 (0.063)	0.90 (0.066)
**Jogging, *n* (%)**	1 107 (6.0)	264 (5.7)	305 (6.6)	294 (6.4)	244 (5.3)

To explore the associations of covariates with the 5 obesity measures, Table 2 shows the results of regressing each obesity measure on sex, age, educational attainment, drinking status, smoking status, regular exercise, and the first 10 PCs. Sex was the most significant predictor for all 5 obesity measures. Except for BFP, males had larger mean values than females in the other 4 obesity measures. Educational attainment and regular exercise were also significant predictors for all 5 metrics. These results were consistent with previous findings: attaining a higher education degree [35] and performing regular physical exercise [46] were associated with a decrease in obesity measures.

**Table 2:** The regression models for the 5 obesity measures (prior to GRS-exercise interaction analysis).

	BMI (kg/m^2^)		Body fat %		Waist circumference (cm)		Hip circumference (cm)		Waist-to-hip ratio	
**Explanatory variables in the regression model** ^1^	Beta	*P*-value	Beta	*P*-value	Beta	*P*-value	Beta	*P*-value	Beta	*P*-value
Sex (1: female vs. 0: male)	-1.846	3.8E-229	8.472 ^2^	0 ^3^	-7.141	0 ^3^	-2.590	5.3E-126	-0.0505	0 ^3^
Age (in years, continuous variable)	-0.001	0.67	0.007	0.17	0.089	6.0E-35	-0.070	9.1E-40	0.0016	1.0E-259
Educational attainment (a value ranging from 1 to 7)	-0.489	9.3E-62	-0.876	3.3E-67	-1.279	1.0E-61	-0.436	8.1E-15	-0.0092	6.2E-79
Drinking status (1: yes vs. 0: no)	0.058	0.58	0.487	6.5E-3	0.702	0.010	-0.031	0.877	0.0078	7.1E-6
Smoking status (1: yes vs. 0: no)	0.165	0.059	0.608	4.8E-5	0.942	4.0E-5	-0.091	0.589	0.0101	4.8E-12
Regular exercise (1: yes vs. 0: no)	-0.286	4.7E-7	-0.813	4.6E-17	-1.242	6.7E-17	-0.644	3.0E-9	-0.0067	1.1E-12
**R-square ^4^**	**7.4%**		**36.0%**		**15.7%**		**4.9%**		**24.7%**	

1. Each obesity measure was regressed on sex, age, educational attainment, drinking status, smoking status, regular exercise, and the first 10 PCs. To save space, we here omit the results of the 10 PCs.

2. Compared with males, females have a greater mean body fat percentage by 8.472%.

3. A *P*-value of “0” is smaller than “1.0E-259”, representing the test is extremely significant.

4. R-square: the proportion of variance in an obesity measure that can be explained by sex, age, educational attainment, drinking status, smoking status, regular exercise, and the first 10 PCs.

### Interactions between GRS and regular physical exercise

Among the 18,424 subjects, 7,652 (41.5%) reported performing regular exercise, while 10,764 reported no regular exercise. A total of 8 subjects did not respond to this question. For a subject who reported performing regular exercise, he/she would then be asked questions regarding the kinds of exercise, the frequency of engaging in a particular exercise per month, and the duration in each practice. An individual could enumerate up to 3 kinds of regular exercise.

**Fig 1:**
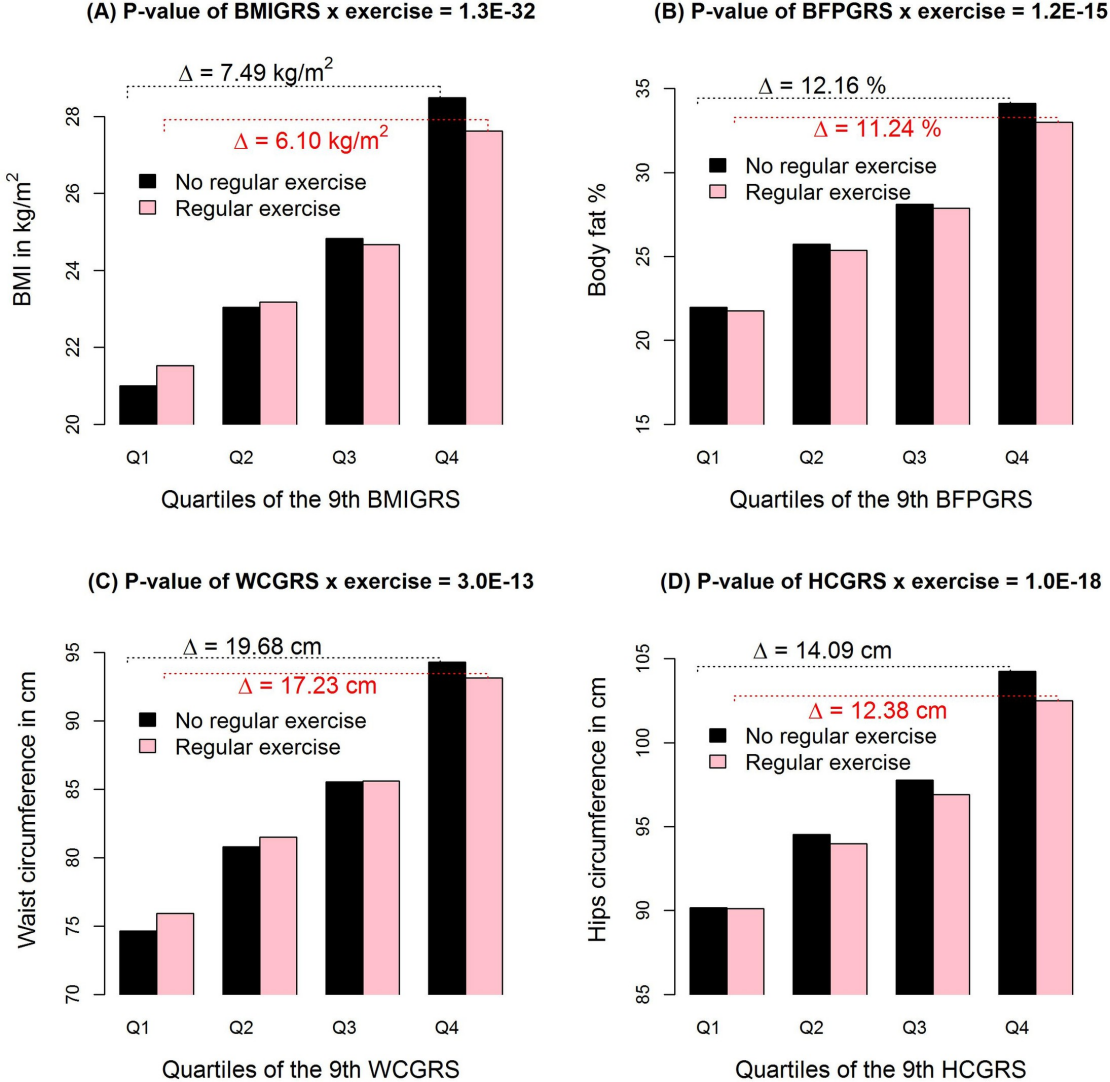
**Average BMI (A), BFP (B), WC (C) and HC (D) stratified by their respective GRS quartiles and regular exercise.** Each plot shows the average of an obesity measure stratified by regular exercise and the quartiles of the 9^th^ GRS, where the marginal-association *P*-value threshold was set at 0.05. We used this GRS for plots because 0.05 is generally considered as the significance level in statistical analyses. The title on each plot is the GRS-M *P*-value that can be found from Table 3. “△” represents the difference in average BMI (A), BFP (B), WC (C) or HC (D) between the top GRS quarter and the bottom GRS quarter. From the plots we can see that the effect of GRS was larger in the physically inactive subjects than in the physically active subjects. The plots for WHR are not presented because the WHRGRS-exercise (*p* = 1) interaction was not significant (Table 3).

Table 3 shows that each 1 s.d. increase in BMIGRS was associated with a 0.43 kg/m^2^ lower BMI in exercisers than in nonexercisers (*p* = 1.3×10^−32^). Each 1 s.d. increase in BFPGRS was associated with a 0.62% lower BFP in exercisers than in nonexercisers (*p* = 1.2×10^−15^, Table 3). Regular physical exercise also significantly attenuated the genetic effects on WC and HC. However, the WHRGRS-exercise interaction was not significant (*p* = 1). Fig 1 shows the average BMI, BFP, WC and HC stratified by GRS quartiles and regular exercise. The effects of GRSs on these 4 obesity measures were smaller in physically active subjects than in physically inactive subjects. Regular exercise attenuated the genetic predisposition to obesity measures.

**Table 3:** Interaction between GRS and exercise on each obesity measure (significant results with *p* < 9.1x10^-5^ are highlighted).

Regular exercise x 5 obesity measures = 5 tests 18 kinds of exercise x 5 obesity measures = 90 tests				BMI (kg/m^2^)		Body fat %		Waist circumference (cm)		Hip circumference (cm)		Waist-to-hip ratio	
	No. of subjects	% of males	Age (years), mean (s.d.)	${\hat{\beta }}_{Int}$	GRS-M *P*-value ^1^	${\hat{\beta }}_{Int}$	GRS-M *P*-value ^1^	${\hat{\beta }}_{Int}$	GRS-M *P*-value ^1^	${\hat{\beta }}_{Int}$	GRS-M *P*-value ^1^	${\hat{\beta }}_{Int}$	GRS-M *P*-value ^1^
**Regular exercise**	7,652	50.9	53.5 (10.3)	**-0.43** ^2^	**1.3E-32** **(4,047)** ^3^	**-0.62**	**1.2E-15** **(865)**	**-0.70**	**3.0E-13** **(3,987)**	**-0.70**	**1.0E-18** **(1,652)**	-0.001	1
Specific analysis for kinds of exercise: Some subjects engage in 2 or 3 kinds of regular exercise. The following 18 kinds of exercise were sorted according to popularity.													
**Walking**	2,637	47.3	55.8 (9.2)	**-0.25**	**5.3E-07** **(7,753)**	-0.15	4.0E-01	-0.52	8.6E-04	-0.30	4.7E-03	0.00293	0.049
**Exercise walking**	1,439	52.3	54.6 (9.3)	**-0.35**	**3.5E-06** **(4,047)**	-0.57	1.2E-04	-0.85	2.0E-03	-0.64	1.7E-04	-0.00266	0.671
**Jogging**	1,107	81.1	45.4 (10.1)	**-0.41**	**1.1E-07** **(7,753)**	**-0.59** ^4^	**7.7E-05** **(4,101)**	-0.68	2.7E-04	**-0.86**	**2.8E-06** **(1,652)**	-0.00382	0.010
**Cycling**	989	68.6	51.4 (10.4)	-0.24	4.4E-01	-0.48	3.8E-02	-0.46	3.4E-01	-0.24	1	-0.00459	0.130
**Mountain climbing**	628	57.3	55.2 (8.2)	**-0.57**	**3.1E-07** **(4,047)**	-0.49	3.5E-03	-0.78	2.7E-03	-0.61	6.8E-04	-0.00387	0.486
**Stretching exercise**	602	33.9	58.1 (8.4)	-0.26	2.5E-01	-0.52	3.3E-01	-0.58	5.4E-01	-0.33	1	-0.00342	0.752
**International standard dancing**	513	13.8	56.8 (7.7)	**-0.43**	**1.8E-05** **(7,753)**	-0.57	1.3E-03	-0.49	2.5E-01	-0.36	2.0E-01	-0.00181	1
**Swimming**	486	66.5	52.7 (10.7)	-0.29	5.3E-01	-0.51	4.4E-01	0.63	2.0E-01	-0.23	1	0.00580	0.172
**Tai Chi**	449	55.7	56.5 (9.1)	-0.60	3.7E-04	-1.09	2.3E-04	-1.01	5.8E-02	-1.03	7.2E-04	-0.00719	0.053
**Dance dance revolution**	420	8.3	50.5 (10.6)	-0.31	7.0E-02	-0.69	1.0E-01	-0.79	1.7E-01	-0.64	1.9E-02	0.00280	0.671
**Yoga**	379	10.3	51.5 (9.8)	-0.74	4.5E-04	0.19	1	-1.23	4.1E-02	-0.75	3.2E-01	0.00250	1
**Qigong**	377	36.3	58.1 (7.8)	-0.39	2.6E-01	-0.28	7.5E-01	-0.71	8.7E-01	-1.08	2.4E-02	-0.00238	1
**Others**	285	41.4	53.5 (11.7)	-0.22	1	-0.59	5.1E-01	-0.87	1	0.64	5.0E-01	-0.00511	0.997
**Weight training**	218	72.9	45.4 (11.3)	-0.33	1.2E-01	-0.63	4.5E-02	-0.82	2.8E-01	-0.47	6.7E-01	0.00333	1
**Badminton**	204	78.9	46.0 (9.5)	-0.28	1	-0.50	1	-0.39	1	-0.57	1	0.00564	1
**Table tennis**	169	76.3	54.1 (10.6)	-0.62	5.4E-02	-0.65	3.3E-01	-0.77	8.1E-01	-0.73	1.3E-01	0.00718	0.916
**Basketball**	119	97.5	40.8 (9.0)	0.40	9.7E-01	-0.81	1	1.12	5.0E-01	-1.29	2.9E-01	-0.00708	0.232
**Tennis**	110	80.9	54.2 (10.0)	-0.39	1	-1.52	7.3E-02	1.85	6.9E-01	0.95	1	-0.00325	1

1. For each obesity measure, 10 GRSs were calculated, and then 10 regression models were fitted. To adjust for multiple testing, the GRS-M *P*-value was reported as 10 times the minimum *P*-value of the 10 GRS-exercise interaction tests.

2. Each 1 s.d. increase in BMIGRS was associated with a 0.43 kg/m^2^ lower BMI in exercisers than in nonexercisers. The regression model was built as BMI = *β*_0_ + *β*_*GRS*_BMIGRS +*β*_*E*_Regular exercise + *β*_*Int*_BMIGRS x Regular exercise + ***β***_*C*_**Covariates** + *ε*. Covariates adjusted in the regression model included sex, age, educational attainment, drinking status, smoking status, and the first 10 PCs. The main effect of regular exercise (${\hat{\beta }}_{E}$) could be found from S8 Table.

3. The significant BMIGRS-exercise interaction was detected at the 8^th^ BMIGRS (the marginal-association *P*-value threshold = 0.025), which included the information of 4,047 SNPs.

4. Each 1 s.d. increase in BFPGRS was associated with a 0.59% lower BFP in joggers than in nonjoggers. The regression model was built as BFP = *β*_0_ + *β*_*GRS*_ BFPGRS + *β*_*E*_Regular jogging + *β*_*Int*_BFPGRS x Regular jogging + ***β***_*C*_**Covariates** + *ε*. Covariates adjusted in the regression model included sex, age, educational attainment, drinking status, smoking status, the first 10 PCs, 17 covariates regarding engaging in the other 17 kinds of exercise or not, and the interaction terms between BFPGRS and the 17 kinds of exercise. The main effect of regular jogging ($\hat{\beta_{E}}$) could be found from S8 Table.

### Interactions between GRS and eighteen kinds of exercise

We then performed a specific analysis for the 18 kinds of exercise. Some TWB individuals reported multiple kinds of regular exercise, and a limit of 3 kinds could be recorded by TWB interviewers. Therefore, when we assessed the interaction between a GRS and a kind of exercise, whether a person also engaged in other kinds of exercise should be considered. The regression models were similar with model (3), but more covariates were adjusted in the models. For example, to investigate the BMIGRS-jogging interaction on BMI, we regressed BMI on a BMIGRS, jogging or not (1: yes vs. 0: no), the interaction between them, while adjusting for sex, age, educational attainment, drinking status, smoking status, the first 10 PCs, 17 covariates regarding engaging in the other 17 kinds of exercise or not, and the 17 BMIGRS-exercise interaction terms.

**Fig 2:**
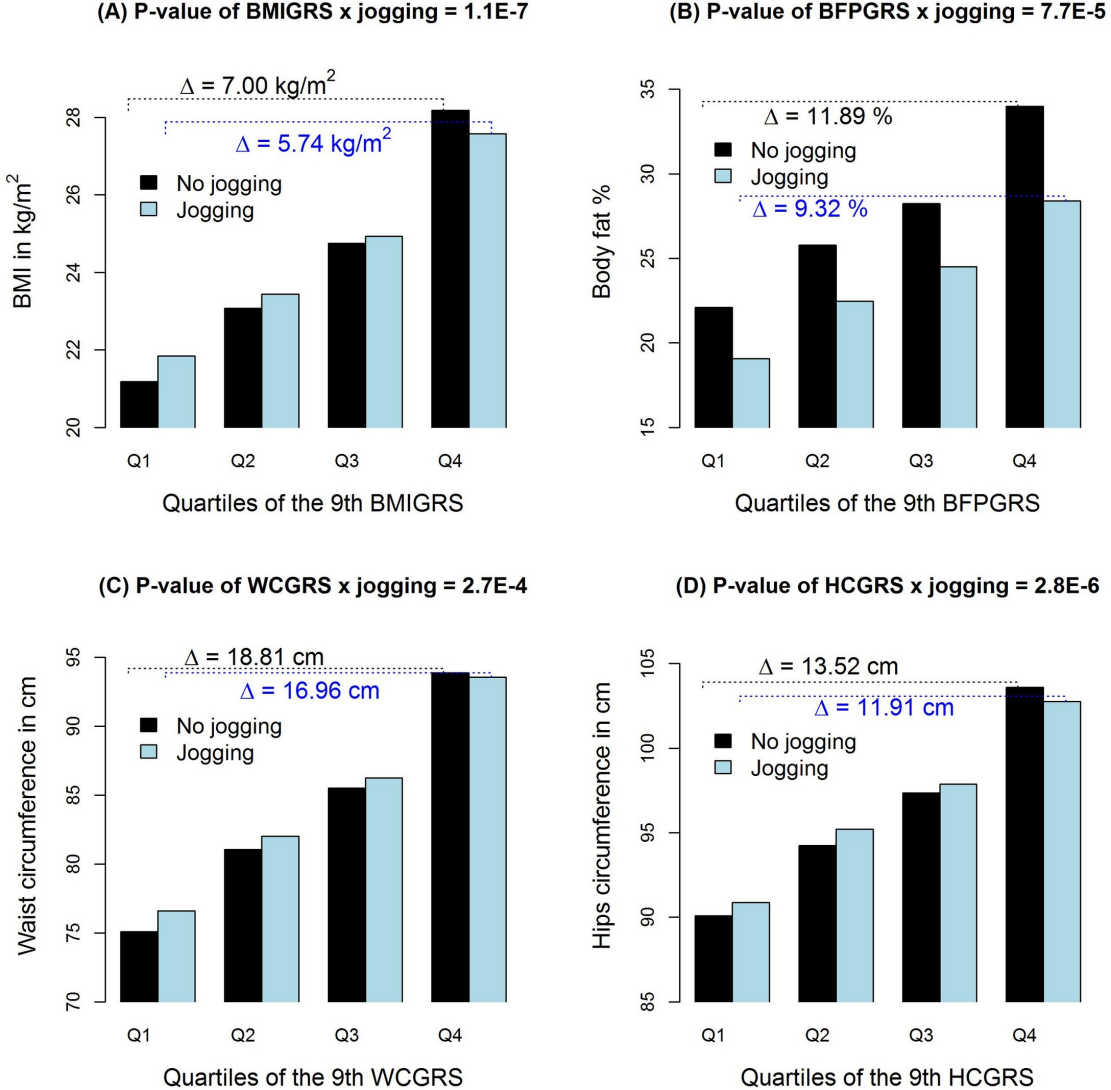
**Average BMI (A), BFP (B), WC (C) and HC (D) stratified by their respective GRS quartiles and jogging.** Each plot shows the average of an obesity measure stratified by jogging and the quartiles of the 9^th^ GRS, where the marginal-association *P*-value threshold was set at 0.05. We used this GRS for plots because 0.05 is generally considered as the significance level in statistical analyses. The title on each plot is the GRS-M *P*-value that can be found from Table 3. “△” represents the difference in average BMI (A), BFP (B), WC (C) or HC (D) between the top GRS quarter and the bottom GRS quarter. From the plots we can see that the effect of GRS was larger in the nonjoggers than in the joggers. The plots for WHR are not presented because the WHRGRS-jogging (*p* = 0.01) interaction was not significant (Table 3).

As shown in Table 3, all types of exercise generally attenuate the genetic contributions of BMI, BFP, WC and HC, as indicated by the direction of the interaction terms (${\hat{\beta }}_{Int}<0$). Among the 18 kinds of exercise, jogging, mountain climbing, walking, exercise walking, and international standard dancing significantly attenuated the genetic effects on BMI (*p* < 9.1x10^-5^). Moreover, jogging additionally attenuated the genetic effects on BFP and HC. As shown in Table 3, across all 5 obesity measures, jogging consistently presented the most significant interactions with GRS (i.e., the smallest *P*-value). Fig 2 shows the average BMI, BFP, WC and HC stratified by GRS quartiles and jogging. The effects of GRSs on these 4 obesity measures were smaller in joggers than in nonjoggers. The results of exercise frequency (Table 4) and duration (Table 5) were similar to those of engaging in the kind of exercise (Table 3). Additionally, a longer practice of yoga could blunt the genetic effects on BMI (Table 5).

**Table 4:** Interaction between GRS and exercise frequency per month (significant results with *p* < 9.1x10^-5^ are highlighted) (18 exercise frequencies x 5 obesity measures = 90 tests).

	Frequency per month		BMI (kg/m^2^)		Body fat %		Waist circumference (cm)		Hip circumference (cm)		Waist-to-hip ratio	
	Mean	Standard deviation	${\hat{\beta }}_{Int}$	GRS-M *P*-value ^1^	${\hat{\beta }}_{Int}$	GRS-M *P*-value ^1^	${\hat{\beta }}_{Int}$	GRS-M *P*-value ^1^	${\hat{\beta }}_{Int}$	GRS-M *P*-value ^1^	${\hat{\beta }}_{Int}$	GRS-M *P*-value ^1^
**Walking**	20.2	9.6	**-0.011**	**1.7E-07** **(7,753)**	-0.009	7.0E-02	-0.021	2.1E-03	-0.015	1.0E-03	0.00008	0.988
**Exercise walking**	18.5	8.8	**-0.022**	**4.9E-06** **(481)**	**-0.030**	**4.8E-05** **(1,638)**	-0.044	4.7E-04	**-0.033**	**3.1E-05** **(1,652)**	-0.00013	0.215
**Jogging**	14.5	7.7	**-0.025** ^2^	**9.4E-08** **(7,753)** ^3^	-0.027	3.5E-03	-0.043	1.9E-02	**-0.052**	**1.6E-06** **(1,652)**	-0.00018	0.343
**Cycling**	15.2	9.8	-0.017	4.6E-02	-0.023	9.0E-02	-0.037	2.6E-01	-0.015	1	-0.00029	0.053
**Mountain climbing**	9.0	7.9	**-0.039**	**6.7E-05** **(4,047)**	-0.032	2.9E-02	-0.048	1.1E-01	-0.034	6.5E-02	-0.00027	0.690
**Stretching exercise**	21.3	8.4	-0.014	1.7E-01	-0.024	2.3E-01	-0.016	1	-0.015	1	-0.00016	0.587
**International standard dancing**	16.3	8.2	-0.022	1.3E-04	-0.027	9.9E-03	-0.029	1.5E-01	-0.026	2.8E-02	0.00017	1
**Swimming**	15.4	9.6	-0.021	1.2E-01	-0.032	2.8E-01	0.027	5.1E-01	-0.029	9.0E-01	0.00034	0.098
**Tai Chi**	18.7	9.5	-0.028	2.5E-03	-0.048	2.3E-03	-0.038	3.0E-01	-0.041	7.6E-03	-0.00030	0.145
**Dance dance revolution**	14.1	7.7	-0.020	3.3E-02	-0.046	8.3E-02	-0.041	4.4E-01	-0.031	1.2E-01	0.00019	0.438
**Yoga**	11.9	8.1	-0.056	1.8E-04	-0.025	1	-0.117	3.0E-03	-0.042	5.7E-02	0.00021	1
**Qigong**	21.4	9.4	-0.009	1	-0.009	1	0.014	1	-0.036	1.3E-01	-0.00007	1
**Others**	18.3	11.9	-0.019	5.1E-01	0.030	4.0E-01	-0.050	4.0E-01	0.015	1	-0.00025	0.998
**Weight training**	15.4	9.1	-0.017	1.4E-01	-0.034	3.6E-02	-0.050	1.0E-01	-0.028	4.0E-01	0.00020	1
**Badminton**	11.4	6.9	-0.030	4.0E-01	-0.071	1.0E-01	-0.057	6.9E-01	-0.044	4.4E-01	-0.00025	1
**Table tennis**	15.7	8.4	-0.036	8.3E-02	-0.033	2.3E-01	-0.028	1	-0.046	5.9E-02	0.00057	0.118
**Basketball**	10.9	7.6	0.024	1	-0.055	1	0.060	1	-0.088	6.5E-01	-0.00056	0.099
**Tennis**	16.7	8.4	-0.035	2.5E-01	-0.089	3.4E-02	0.077	1	-0.023	1	0.00029	1

1. For each obesity measure, 10 GRSs were calculated, and then 10 regression models were fitted. To adjust for multiple testing, the GRS-M *P*-value was reported as 10 times the minimum *P*-value of the 10 GRS-exercise interaction tests.

2. Each 1 s.d. increase in BMIGRS was associated with a 0.025 kg/m^2^ lower BMI in subjects having 1 more jog per month. The regression model was built as BMI = *β*_0_ + *β*_*GRS*_BMIGRS + *β*_*E*_Jogging frequency + *β*_*Int*_BMIGRS x Jogging frequency + ***β***_*C*_**Covariates** + *ε*. Covariates adjusted in the regression model included sex, age, educational attainment, drinking status, smoking status, the first 10 PCs, 17 covariates regarding the frequencies per month of the other 17 kinds of exercise, and the interaction terms between BMIGRS and the frequencies of the 17 kinds of exercise. For subjects who did not engage in jogging, their jogging frequencies were coded as 0. The main effect of jogging frequency (${\hat{\beta }}_{E}$) could be found from S9 Table.

3. The significant interaction between BMIGRS and jogging frequency per month was detected at the 9^th^ BMIGRS (marginal-association *P*-value threshold = 0.05), which included the information of 7,753 SNPs.

**Table 5:** Interaction between GRS and exercise duration (in hours) (significant results with *p* < 9.1x10^-5^ are highlighted) (18 exercise durations x 5 obesity measures = 90 tests).

	Duration for each exercise (hours)		BMI (kg/m^2^)		Body fat %		Waist circumference (cm)		Hip circumference (cm)		Waist-to-hip ratio	
	Mean	Standard deviation	${\hat{\beta }}_{Int}$	GRS-M *P*-value ^1^	${\hat{\beta }}_{Int}$	GRS-M *P*-value ^1^	${\hat{\beta }}_{Int}$	GRS-M *P*-value ^1^	${\hat{\beta }}_{Int}$	GRS-M *P*-value ^1^	${\hat{\beta }}_{Int}$	GRS-M *P*-value ^1^
**Walking**	0.78	0.39	**-0.26**	**5.4E-06** **(7,753)**	-0.13	6.9E-01	-0.60	5.6E-04	-0.33	6.5E-03	0.00365	0.019
**Exercise walking**	0.81	0.38	**-0.35**	**4.1E-05** **(4,047)**	-0.63	1.9E-04	-0.78	1.2E-02	-0.67	1.6E-04	-0.00289	0.596
**Jogging**	0.70	0.33	**-0.60**	**3.9E-08** **(4,047)**	**-0.84**	**4.4E-06** **(4,101)**	-0.79	9.0E-04	**-1.01**	**2.1E-05** **(1,652)**	-0.00417	0.034
**Cycling**	1.16	0.89	-0.06	1	-0.26	3.6E-01	-0.34	2.6E-01	-0.24	1	-0.00205	0.734
**Mountain climbing**	1.99	1.22	**-0.24**	**1.9E-06** **(4,047)**	-0.20	9.9E-03	-0.38	4.1E-04	-0.25	3.3E-03	-0.00148	0.103
**Stretching exercise**	0.73	0.36	-0.29	4.3E-01	-0.50	9.0E-01	-0.75	3.8E-01	-0.30	1	-0.00358	1
**International standard dancing**	1.28	0.61	**-0.28**	**9.1E-05** **(7,753)**	-0.38	1.3E-03	-0.35	2.9E-01	-0.22	3.6E-01	-0.00128	1
**Swimming**	0.84	0.50	-0.15	1	-0.24	1	0.51	4.9E-01	-0.34	7.0E-01	0.00577	0.203
**Tai Chi**	1.13	0.54	-0.36	3.0E-03	-0.77	1.5E-03	-0.91	1.8E-02	-0.83	5.4E-04	-0.00395	0.599
**Dance dance revolution**	1.01	0.43	-0.22	3.1E-01	-0.45	5.3E-01	-0.67	2.4E-01	-0.48	8.6E-02	0.00226	1
**Yoga**	1.17	0.49	**-0.66** ^2^	**5.3E-05** **(481)** ^3^	-0.33	1	-1.10	1.1E-02	-0.71	8.5E-02	0.00230	0.566
**Qigong**	1.01	0.46	-0.33	1.8E-01	-0.53	1.8E-01	-0.61	9.9E-01	-0.99	3.1E-02	-0.00163	1
**Others**	0.96	0.65	-0.31	1	-0.58	4.2E-01	-0.95	3.1E-01	0.29	1	-0.00567	0.349
**Weight training**	0.80	0.48	-0.30	2.6E-01	-0.41	7.7E-01	-1.18	4.7E-02	-0.49	4.3E-01	-0.00556	1
**Badminton**	1.40	0.60	-0.22	1	-0.41	8.4E-01	-0.21	1	-0.33	1	0.00437	1
**Table tennis**	1.34	0.59	-0.40	1.3E-01	-0.42	3.4E-01	-0.43	1	-0.60	1.1E-01	0.00365	1
**Basketball**	1.40	0.68	0.32	3.5E-01	-0.60	7.8E-01	0.74	3.7E-01	-0.97	2.0E-01	-0.00626	1
**Tennis**	1.41	0.64	-0.40	4.7E-01	-1.09	2.6E-02	0.68	1	0.60	1	-0.00330	1

1. For each obesity measure, 10 GRSs were calculated, and then 10 regression models were fitted. To adjust for multiple testing, the GRS-M *P*-value was reported as 10 times the minimum *P*-value of the 10 GRS-exercise interaction tests.

2. Each 1 s.d. increase in BMIGRS was associated with a 0.66 kg/m^2^ lower BMI in subjects with 1 more hour in each yoga practice. The regression model was built as BMI = *β*_0_ + *β*_*GRS*_ BMIGRS + *β*_*E*_ Yoga duration + *β*_*Int*_ BMIGRS x Yoga duration + ***β***_*C*_**Covariates** + *ε*. Covariates adjusted in the regression model included sex, age, educational attainment, drinking status, smoking status, the first 10 PCs, 17 covariates regarding the duration (in hours) of the other 17 kinds of exercise, and the interaction terms between BMIGRS and the duration of the 17 kinds of exercise. For subjects who did not choose yoga as their regular exercise, their yoga duration was coded as 0. The main effect of yoga duration (${\hat{\beta }}_{E}$) could be found from S10 Table.

3. The significant interaction between BMIGRS and the duration in each yoga practice was detected at the 5^th^ BMIGRS (marginal-association *P*-value threshold = 0.0025), which included the information of 481 SNPs.

**Fig 3:**
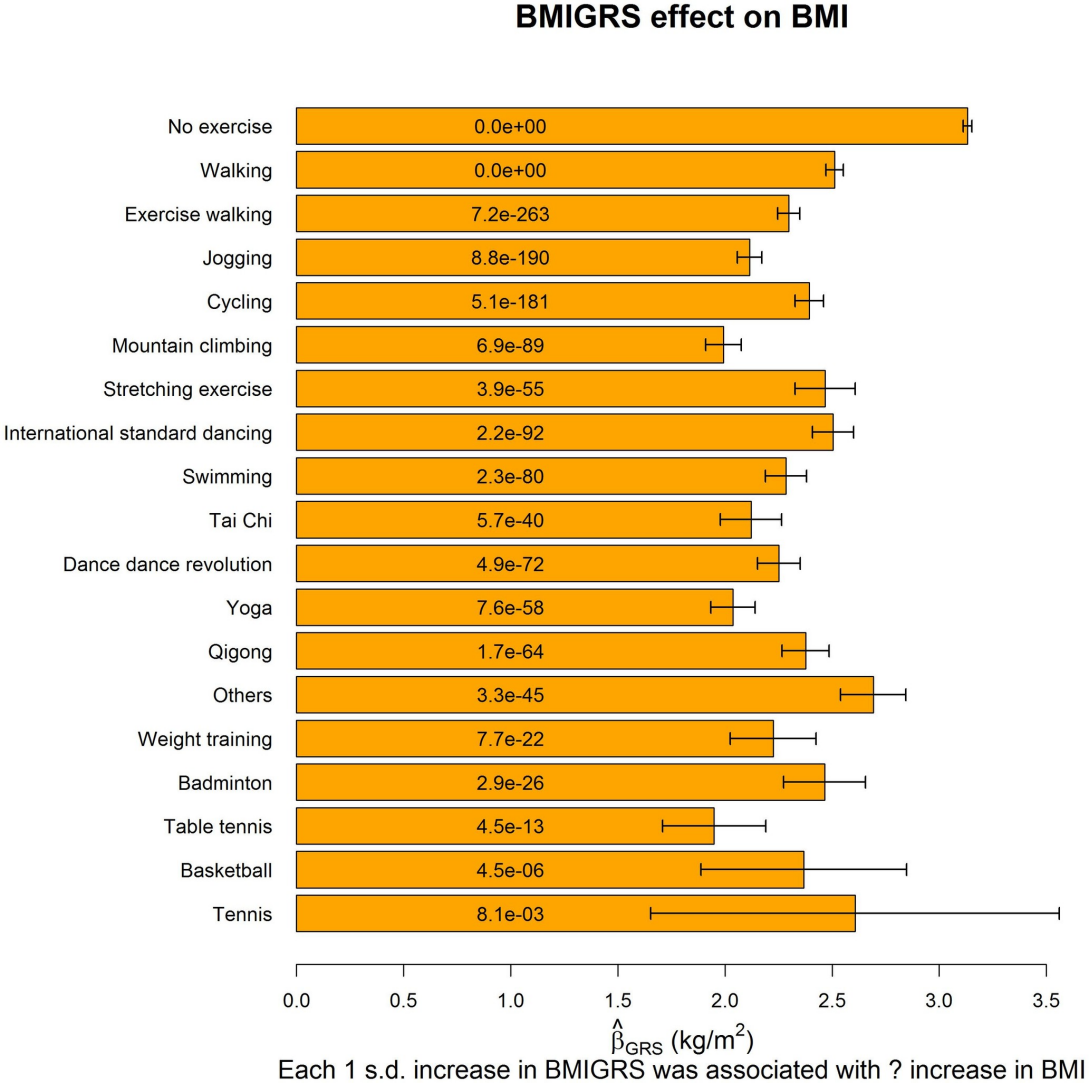
The effect of BMIGRS on BMI. The regression model (stratified by exercise types) was built as *BMI* = *β*_0_ + *β*_*GRS*_ *BMIGRS* + ***β***_***C***_***Covariates*** + *ε*, where *BMIGRS* was calculated at the marginal-association *P*-value threshold of 0.05. We used this BMIGRS for plots because 0.05 is generally considered as the significance level in statistical analyses. The orange bars represent ${\hat{\beta }}_{GRS}$ on BMI (stratified by exercise types), and the black segments mark $\left[{\hat{\beta }}_{GRS}-standard\;error\;of\;{\hat{\beta }}_{GRS},\;{\hat{\beta }}_{GRS}+standard\;error\;of\;{\hat{\beta }}_{GRS}\right]$. The text on each bar is the *P*-value of testing *H*_0_: *β*_*GRS*_ = 0 vs. *H*_1_: *β*_*GRS*_ ≠ 0. Covariates adjusted in the regression model included sex, age, educational attainment, drinking status, smoking status, and the first 10 PCs. Consistent with Table 3, the 18 kinds of exercise were sorted according to popularity.

Fig 3 shows the effect of BMIGRS on BMI, stratified by exercise types. All types of exercise generally attenuate the genetic effects of BMI, as indicated by ${\hat{\beta }}_{GRS}$ of each exercise type < ${\hat{\beta }}_{GRS}$ of no exercise. The GRS effects on other 4 obesity measures can be found from S1–S4 Figs.

S11–S13 Tables present the results of GRS×exercise interactions, stratified by sex. The directions of ${\hat{\beta }}_{Int}s$ were in line with the results in Tables 3–5 where sex was treated as a covariate adjusted in model (3). All types of exercise generally attenuate the genetic contributions of BMI, BFP, WC and HC, as indicated by the direction of the interaction terms (${\hat{\beta }}_{Int}<0$).

## Discussion

Obesity is a major global public health problem, especially in developed countries [47]. Obesity is complicated as it is caused by an interplay of multiple genes and lifestyle factors [9]. Numerous studies have reported that the effects of a BMIGRS are larger in physically inactive subjects than in physically active subjects [13–15, 18, 20]. However, most of these studies focused on only BMI, without discussing central obesity. Moreover, it remains unknown what kinds of exercise could more effectively blunt the genetic effects on obesity measures. We here used the GRS-M approach [36] to investigate interactions between GRSs and 18 kinds of exercise on 5 commonly used obesity measures.

### Method of G×E analysis

Because 95% of the subjects in Locke *et al*.’s study [34] were of European descent, building GRS according to these 97 SNPs may not be appropriate for other ethnic populations. Although the same data set is used to estimate *β*_*SNP*,*i*_ (*i* = 1,⋯,142040) and to test the significance of *GRS*×*E*, this GRS-M approach is valid in the sense that the type I error rates are satisfactorily controlled [36]. Corollary 1 of Dai *et al*. [43] has justified the validity of using marginal associations (between SNP and an obesity measure) as the filtering test statistics, and the data-splitting strategy is not required. Building GRS with internal weights has been used in some G×E analyses [36–40, 48].

Previous G×E analyses have typically constructed a GRS using SNPs that reached the genome-wide significance level (i.e., *p* < 5×10^−8^) [13–24]. However, some studies have suggested that a GRS comprising more SNPs can improve the prediction for a phenotype [41, 49–51]. SNPs that interact with an environmental factor may not necessarily present a strong marginal association with the phenotype. To explore G×E, it is worthwhile to consider a more liberal threshold than the genome-wide significance level (5×10^−8^). For example, the “Set-Based gene-EnviRonment InterAction test” (SBERIA) constructs a GRS by using all SNPs with a marginal-association *P*-value < 0.1 [39, 40]. In fact, the optimal filtering *P*-value threshold varies with environmental factors and phenotypes [52].

Therefore, the GRS-M method considers 10 *P*-value thresholds for marginal-association filtering [36]. For each obesity measure, 10 GRSs were calculated, and then 10 regression models were fitted. To adjust for multiple testing, the GRS-M *P*-value was reported as 10 times the minimum *P*-value of the 10 GRS-exercise interaction tests. The GRS-M test is a valid statistical method by controlling type I error rates well [36]. As summarized in S7 Table, significant GRS-exercise interactions were detected at a marginal-association *P*-value threshold between 0.0025 and 0.05, and the number of SNPs used to construct each of the GRSs ranged from 481 to 7,753. With the development of relatively inexpensive SNP arrays, using more SNPs than those achieving the genome-wide significance level is currently feasible [53].

### Main findings

Previous studies have found that performing regular physical exercise could blunt the genetic effects on BMI [13–16, 18, 20, 24]. However, few studies have investigated BFP or measures of central obesity. These obesity measures are even more relevant to health than BMI. For example, central obesity is considered to be a predominant risk factor for metabolic syndrome [54, 55]. We here show that performing regular exercise attenuates the genetic effects on 4 obesity measures, including BMI, BFP, WC, and HC (Table 3).

Regarding exercise types, regular jogging mitigated the genetic effects on BMI, BFP, and HC. Mountain climbing, walking, exercise walking, and international standard dancing also attenuated the genetic effects on BMI (Table 3). Moreover, a longer practice of yoga blunted the genetic effects on BMI (Table 5). These results indicated that although hereditary factors are critical to obesity, performing different kinds of exercise can modify this relationship to various extents.

A BMI that is too high or too low is associated with an increased mortality rate. According to studies from western Europe and North America [56], a BMI ranging from 22.5 to 25 kg/m^2^ corresponded to the lowest overall mortality. Fig 1(A) shows that regular physical exercise was associated with an increase in BMI at a low BMIGRS (the bottom quarter: Q1) but a decrease in BMI at a high BMIGRS (the top quarter: Q4). Performing regular exercise was associated with a reduced risk of having a too-high or a too-low BMI.

Summarizing Tables 3–5, a total of 12 kinds of exercise did not achieve significance for the attenuation of the genetic risk of obesity measures. Plausible reasons included (1) less popularity or (2) a smaller GRS-exercise interaction effect. Exercises such as cycling (989 subjects), stretching exercise (602 subjects), swimming (486 subjects), DDR (420 subjects), and qigong (377 subjects) were more popular or as popular as yoga (379 subjects), but their evidence of interacting with GRS was relatively weak (Table 3). These 5 kinds of exercise may have limited effects on mitigating the genetic risk of obesity measures. In contrast, although the evidence of GRS-Tai Chi interactions did not achieve the Bonferroni-corrected significance level (9.1x10^-5^), the small *P*-values implied that engaging in Tai Chi (449 subjects) might potentially blunt the genetic effects on obesity measures.

Few studies have investigated the interplay between particular kinds of exercise and genetic risk of obesity measures. Therefore, we can hardly compare our results with previous findings. We here provide possible explanations for these results. Cycling (989 subjects), stretching exercise (602 subjects), and qigong (377 subjects) usually require less energy expenditure than the 6 exercises that demonstrate interactions with GRS [57]. Exercises in cold water such as swimming (486 subjects) can especially stimulate appetite and food intake [58, 59]. DDR (420 subjects), a computer game based on dancing with music videos, is not as formal as international standard dancing. These reasons may possibly explain why these 5 popular exercises (cycling, stretching exercise, qigong, swimming, and DDR) cannot mitigate genetic susceptibility to obesity measures.

Because relatively few subjects engaged in weight training (218 subjects), badminton (204 subjects), table tennis (169 subjects), basketball (119 subjects), or tennis (110 subjects), the statistical power to detect the interplay between GRS and these exercises was limited. Further research on these 5 kinds of exercise will be interesting.

### Comparison between our findings and previous studies

A G×E study for BMI using 362,496 UK Biobank subjects has reported that a quicker walking pace attenuated the genetic effects on BMI (the top row in Tables 2–3 of [14]). This is consistent with our findings in Tables 3–5, i.e., $\left|{\hat{\beta }}_{Int}\right|$ of BMIGRS×jogging > $\left|{\hat{\beta }}_{Int}\right|$ of BMIGRS×exercise walking > $\left|{\hat{\beta }}_{Int}\right|$ of BMIGRS×walking. Because pace of jogging > pace of exercise walking > pace of walking, our results also show that a quicker walking pace could more effectively attenuate the genetic effects on BMI. Moreover, the frequency of stair climbing in last 4 weeks has been found to blunt the effect of BMIGRS (Tables 2–3 of [14]). Similarly, we here detected significant interactions between BMIGRS and both the frequency (Table 4) and duration (Table 5) of mountain climbing.

### Associations of 18 kinds of exercise with obesity measures (Main effects of exercises)

Some previous studies investigated the efficacy of performing several kinds of exercise in preventing obesity [60, 61]. For example, a randomized controlled trial with 64 subjects assigned to the Tai Chi group and 78 assigned to the control group demonstrated that performing Tai Chi led to a marked but non-significant reduction in WC [60]. For comparison, in S8–S10 Tables, we listed the associations of 18 kinds of exercise with obesity measures, i.e., ${\hat{\beta }}_{E}$ estimated from model (3). Our results showed that performing Tai Chi was significantly associated with a reduction in WC and BFP (*p* < 9.1x10^-5^). Regular jogging, performing yoga and Tai Chi were associated with a decrease in multiple obesity measures. Moreover, playing table tennis was associated with a reduction in WHR. WC and WHR are indicators of central obesity [8]. Our results show that performing Tai Chi or playing table tennis was related to a reduced risk of central obesity, presumably because waist turning is frequently required when engaging in these two kinds of exercise.

The results for associations of 18 kinds of exercise with obesity measures were robust to the exclusion of GRS and GRS-exercise interaction terms. In addition to obtaining $\hat{\beta_{E}}$ from model (3), we additionally fitted the following model without GRS and the relevant interaction terms:

$$BMI\;(or\;another\;obesity\;measure)=\beta_{0}+\beta_{E}E+\beta_{C}Covariates+\varepsilon ,$$ (4)

where *E* was some kind of exercise, and covariates included sex, age, educational attainment, drinking status, smoking status, the first 10 PCs, and 17 covariates regarding engaging in the other 17 kinds of exercise or not. The results were similar to those obtained from model (3), i.e., regular jogging, performing yoga, Tai Chi and playing table tennis were associated with a decrease in obesity measures.

To sum up, regular jogging and performing yoga were not only associated with a decrease in obesity measures, but they also attenuated the genetic predisposition to obesity measures. Exercises such as walking, exercise walking, mountain climbing, and international standard dancing, were not significantly associated with a change in obesity measures, but these 4 kinds of exercise could blunt the genetic effects on BMI. By comparing rows of “walking” and “yoga” in S8–S10 Tables, our result is consistent with a previous finding that engaging in yoga shows a larger reduction in BMI than walking [61].

It is interesting that, across all 5 obesity measures, regular jogging consistently presented the most significant interactions with GRSs (Table 3). The genetic effects on obesity measures can be decreased to various extents by performing different kinds of exercise. The benefits of regular physical exercise, especially jogging, are more impactful in subjects who are more predisposed to obesity.

## Supporting information

S1 FigThe effect of BFPGRS on BFP.The regression model (stratified by exercise types) was built as *BFP* = *β*_0_ + *β*_*GRS*_*BFPGRS* + ***β***_***C***_***Covariates*** + *ε*, where *BFPGRS* was calculated at the marginal-association *P*-value threshold of 0.05. We used this BFPGRS for plots because 0.05 is generally considered as the significance level in statistical analyses. The orange bars represent ${\hat{\beta }}_{GRS}$ on BFP (stratified by exercise types), and the black segments mark $\left[{\hat{\beta }}_{GRS}-standard\;error\;of\;{\hat{\beta }}_{GRS},\;{\hat{\beta }}_{GRS}+standard\;error\;of\;{\hat{\beta }}_{GRS}\right]$. The text on each bar is the *P*-value of testing *H*_0_: *β*_*GRS*_ = 0 vs. *H*_1_: *β*_*GRS*_ ≠ 0. Covariates adjusted in the regression model included sex, age, educational attainment, drinking status, smoking status, and the first 10 PCs. Consistent with Table 3, the 18 kinds of exercise were sorted according to popularity.(JPG)

S2 FigThe effect of WCGRS on WC.The regression model (stratified by exercise types) was built as *WC* = *β*_0_ + *β*_*GRS*_*WCGRS* + ***β***_***C***_***Covariates*** + *ε*, where *WCGRS* was calculated at the marginal-association *P*-value threshold of 0.05. We used this WCGRS for plots because 0.05 is generally considered as the significance level in statistical analyses. The orange bars represent ${\hat{\beta }}_{GRS}$ on WC (stratified by exercise types), and the black segments mark $\left[{\hat{\beta }}_{GRS}-standard\;error\;of\;{\hat{\beta }}_{GRS},\;{\hat{\beta }}_{GRS}+standard\;error\;of\;{\hat{\beta }}_{GRS}\right]$. The text on each bar is the *P*-value of testing *H*_0_: *β*_*GRS*_ = 0 vs. *H*_1_: *β*_*GRS*_ ≠ 0. Covariates adjusted in the regression model included sex, age, educational attainment, drinking status, smoking status, and the first 10 PCs. Consistent with Table 3, the 18 kinds of exercise were sorted according to popularity.(JPG)

S3 FigThe effect of HCGRS on HC.The regression model (stratified by exercise types) was built as *HC* = *β*_0_ + *β*_*GRS*_*HCGRS* + ***β***_***C***_***Covariates*** + *ε*, where *HCGRS* was calculated at the marginal-association *P*-value threshold of 0.05. We used this HCGRS for plots because 0.05 is generally considered as the significance level in statistical analyses. The orange bars represent ${\hat{\beta }}_{GRS}$ on HC (stratified by exercise types), and the black segments mark $\left[{\hat{\beta }}_{GRS}-standard\;error\;of\;{\hat{\beta }}_{GRS},\;{\hat{\beta }}_{GRS}+standard\;error\;of\;{\hat{\beta }}_{GRS}\right]$. The text on each bar is the *P*-value of testing *H*_0_: *β*_*GRS*_ = 0 vs. *H*_1_: *β*_*GRS*_ ≠ 0. Covariates adjusted in the regression model included sex, age, educational attainment, drinking status, smoking status, and the first 10 PCs. Consistent with Table 3, the 18 kinds of exercise were sorted according to popularity.(JPG)

S4 FigThe effect of WHRGRS on WHR.The regression model (stratified by exercise types) was built as *WHR* = *β*_0_ + *β*_*GRS*_*WHRGRS* + ***β***_***C***_***Covariates*** + *ε*, where *WHRGRS* was calculated at the marginal-association *P*-value threshold of 0.05. We used this WHRGRS for plots because 0.05 is generally considered as the significance level in statistical analyses. The orange bars represent ${\hat{\beta }}_{GRS}$ on WHR (stratified by exercise types), and the black segments mark $\left[{\hat{\beta }}_{GRS}-standard\;error\;of\;{\hat{\beta }}_{GRS},\;{\hat{\beta }}_{GRS}+standard\;error\;of\;{\hat{\beta }}_{GRS}\right]$. The text on each bar is the *P*-value of testing *H*_0_: *β*_*GRS*_ = 0 vs. *H*_1_: *β*_*GRS*_ ≠ 0. Covariates adjusted in the regression model included sex, age, educational attainment, drinking status, smoking status, and the first 10 PCs. Consistent with Table 3, the 18 kinds of exercise were sorted according to popularity.(JPG)

S1 Table The associations of 5 obesity measures with 97 BMI-associated SNPs identified from Europeans (only 86 are polymorphic in TWB subjects). (XLSX)

S2 Table The association of European-based GRS with the 5 obesity measures. (DOCX)

S3 Table Interaction between EuGRS and exercise on each obesity measure. (DOCX)

S4 Table Interaction between EuGRS and exercise frequency per month. (DOCX)

S5 Table Interaction between EuGRS and exercise duration (in hours). (DOCX)

S6 Table The cumulative variance explained by the 86 European BMI-associated SNPs. (DOCX)

S7 Table The numbers of SNPs used to form the GRSs under 10 *P*-value thresholds. (DOCX)

S8 Table Main associations of exercises with obesity measures (significant results with *p* < 9.1x10^-5^ are highlighted). (DOCX)

S9 Table Main associations of exercise frequencies per month with obesity measures (significant results with *p* < 9.1x10^-5^ are highlighted). (DOCX)

S10 Table Main associations of the exercise duration (in hours) with obesity measures (significant results with *p* < 9.1x10^-5^ are highlighted). (DOCX)

S11 Table Interaction between GRS and exercise on each obesity measure (stratified by sex). (DOCX)

S12 Table Interaction between GRS and exercise frequency per month (stratified by sex). (DOCX)

S13 Table Interaction between GRS and exercise duration (in hours) (stratified by sex). (DOCX)
