# Supplementary figures and images for: Performing different kinds of physical exercise differentially attenuates the genetic effects on obesity measures: Evidence from 18,424 Taiwan Biobank participants
Source: PLoS Genet. 2019 Aug 1;15(8):e1008277. doi: 10.1371/journal.pgen.1008277 (PMC6675047; doi:10.1371/journal.pgen.1008277)

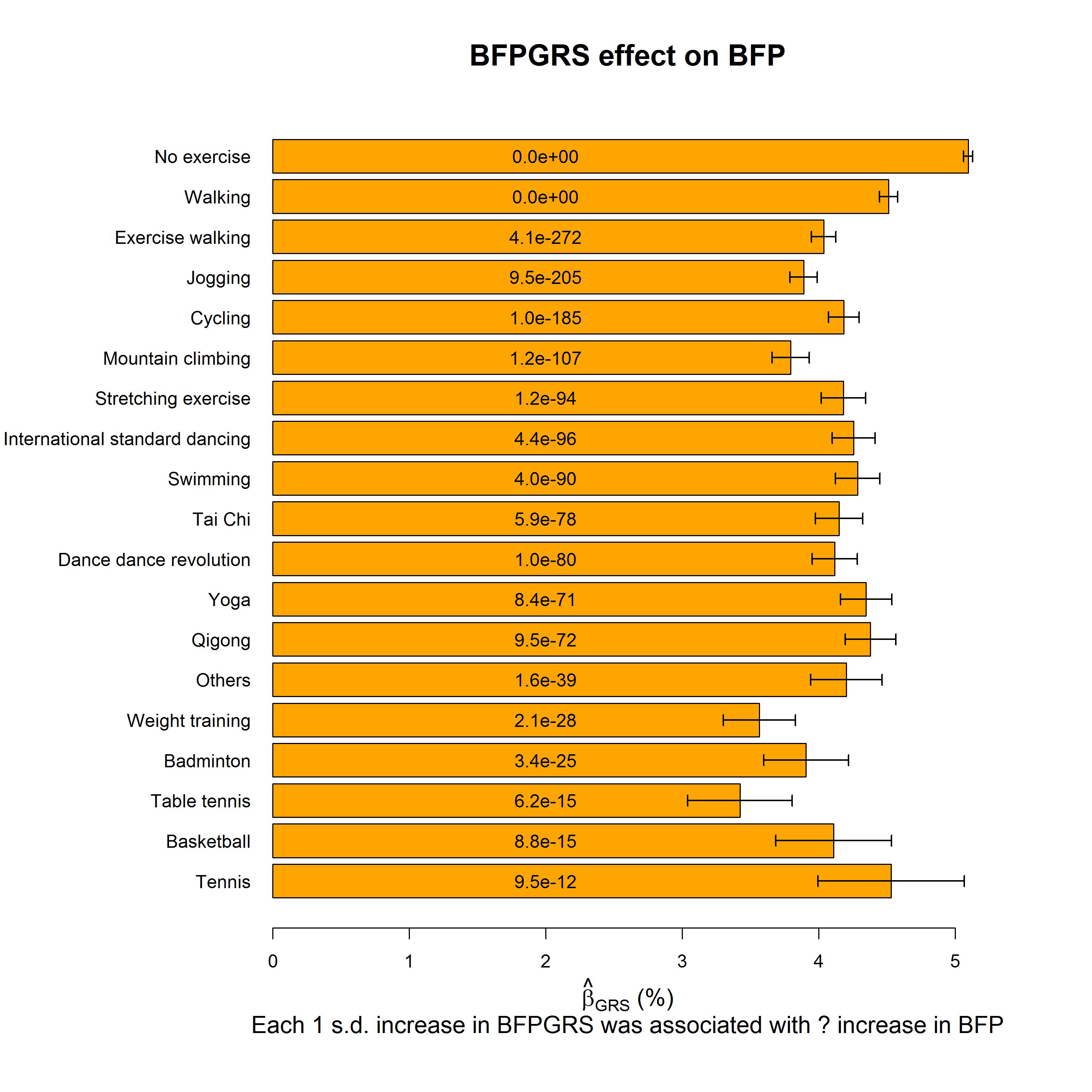

Supplement: S1 Fig — The regression model (stratified by exercise types) was built as BFP = β0 + βGRSBFPGRS + βCCovariates + ε, where BFPGRS was calculated at the marginal-association P-value threshold of 0.05. We used this BFPGRS for plots because 0.05 is generally considered as the significance level in statistical analyses. The orange bars represent β^GRS on BFP (stratified by exercise types), and the black segments mark [β^GRS−standarderrorofβ^GRS,β^GRS+standarderrorofβ^GRS]. The text on each bar is the P-value of testing H0: βGRS = 0 vs. H1: βGRS ≠ 0. Covariates adjusted in the regression model included sex, age, educational attainment, drinking status, smoking status, and the first 10 PCs. Consistent with Table 3, the 18 kinds of exercise were sorted according to popularity. (JPG) [file pgen.1008277.s001.jpg]

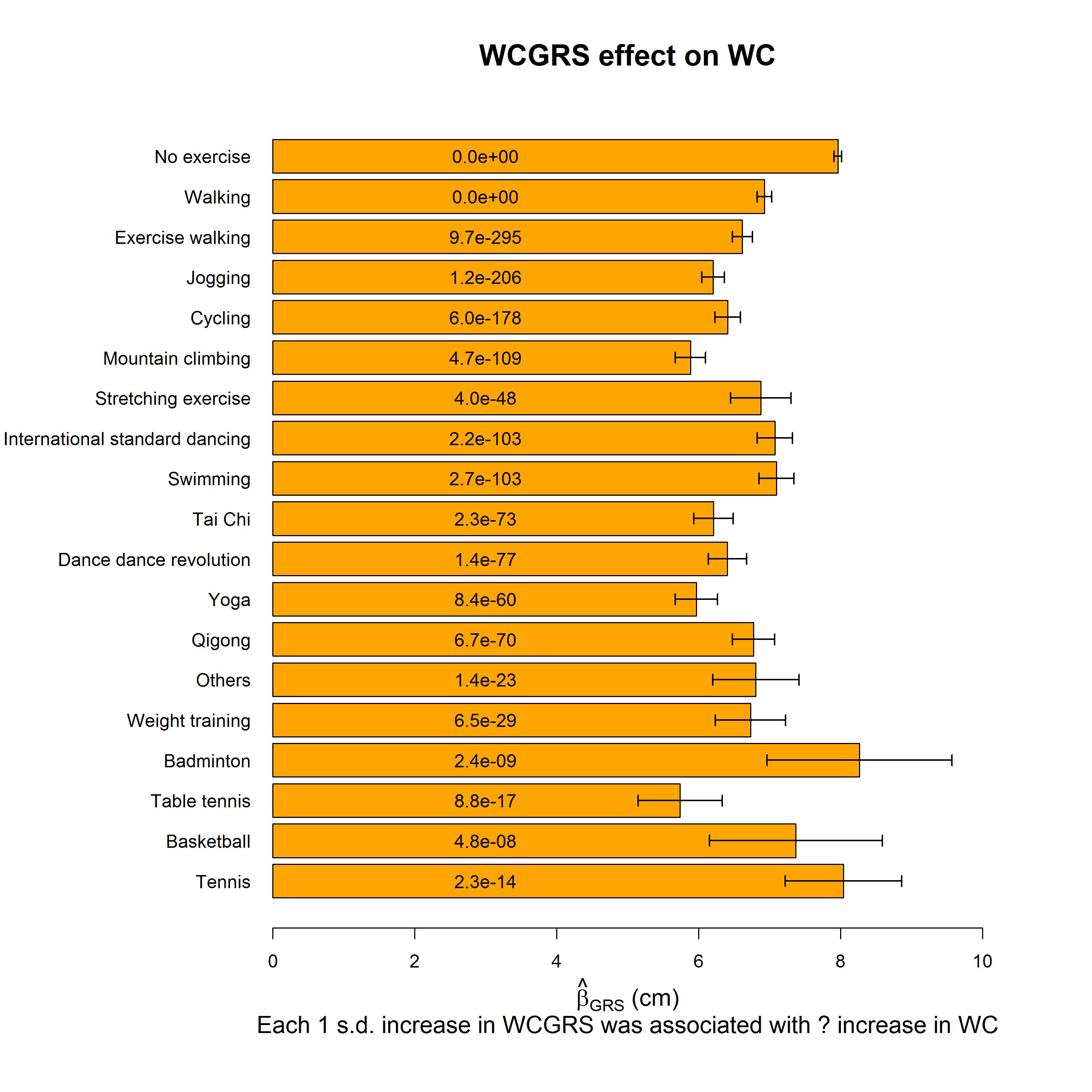

Supplement: S2 Fig — The regression model (stratified by exercise types) was built as WC = β0 + βGRSWCGRS + βCCovariates + ε, where WCGRS was calculated at the marginal-association P-value threshold of 0.05. We used this WCGRS for plots because 0.05 is generally considered as the significance level in statistical analyses. The orange bars represent β^GRS on WC (stratified by exercise types), and the black segments mark [β^GRS−standarderrorofβ^GRS,β^GRS+standarderrorofβ^GRS]. The text on each bar is the P-value of testing H0: βGRS = 0 vs. H1: βGRS ≠ 0. Covariates adjusted in the regression model included sex, age, educational attainment, drinking status, smoking status, and the first 10 PCs. Consistent with Table 3, the 18 kinds of exercise were sorted according to popularity. (JPG) [file pgen.1008277.s002.jpg]

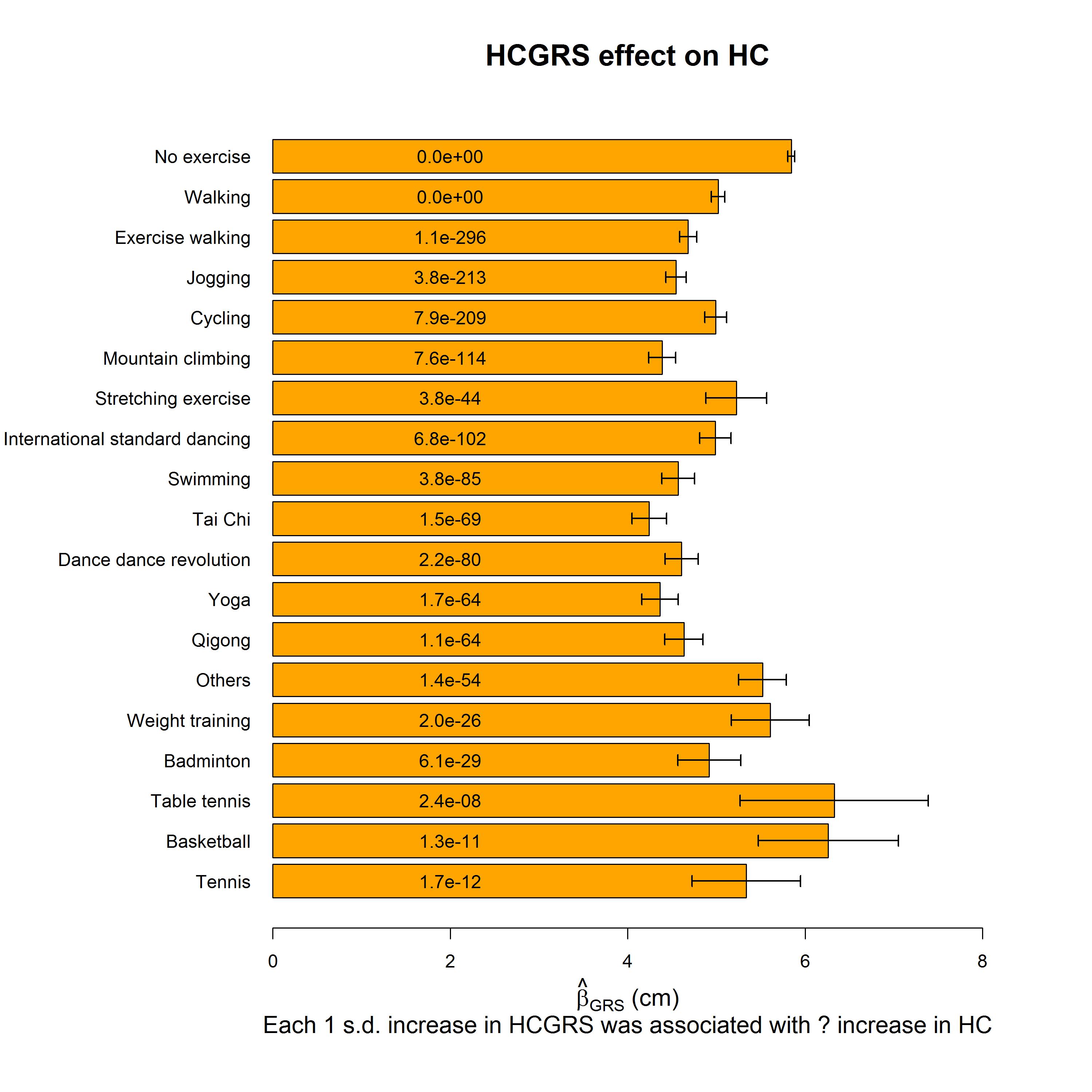

Supplement: S3 Fig — The regression model (stratified by exercise types) was built as HC = β0 + βGRSHCGRS + βCCovariates + ε, where HCGRS was calculated at the marginal-association P-value threshold of 0.05. We used this HCGRS for plots because 0.05 is generally considered as the significance level in statistical analyses. The orange bars represent β^GRS on HC (stratified by exercise types), and the black segments mark [β^GRS−standarderrorofβ^GRS,β^GRS+standarderrorofβ^GRS]. The text on each bar is the P-value of testing H0: βGRS = 0 vs. H1: βGRS ≠ 0. Covariates adjusted in the regression model included sex, age, educational attainment, drinking status, smoking status, and the first 10 PCs. Consistent with Table 3, the 18 kinds of exercise were sorted according to popularity. (JPG) [file pgen.1008277.s003.jpg]

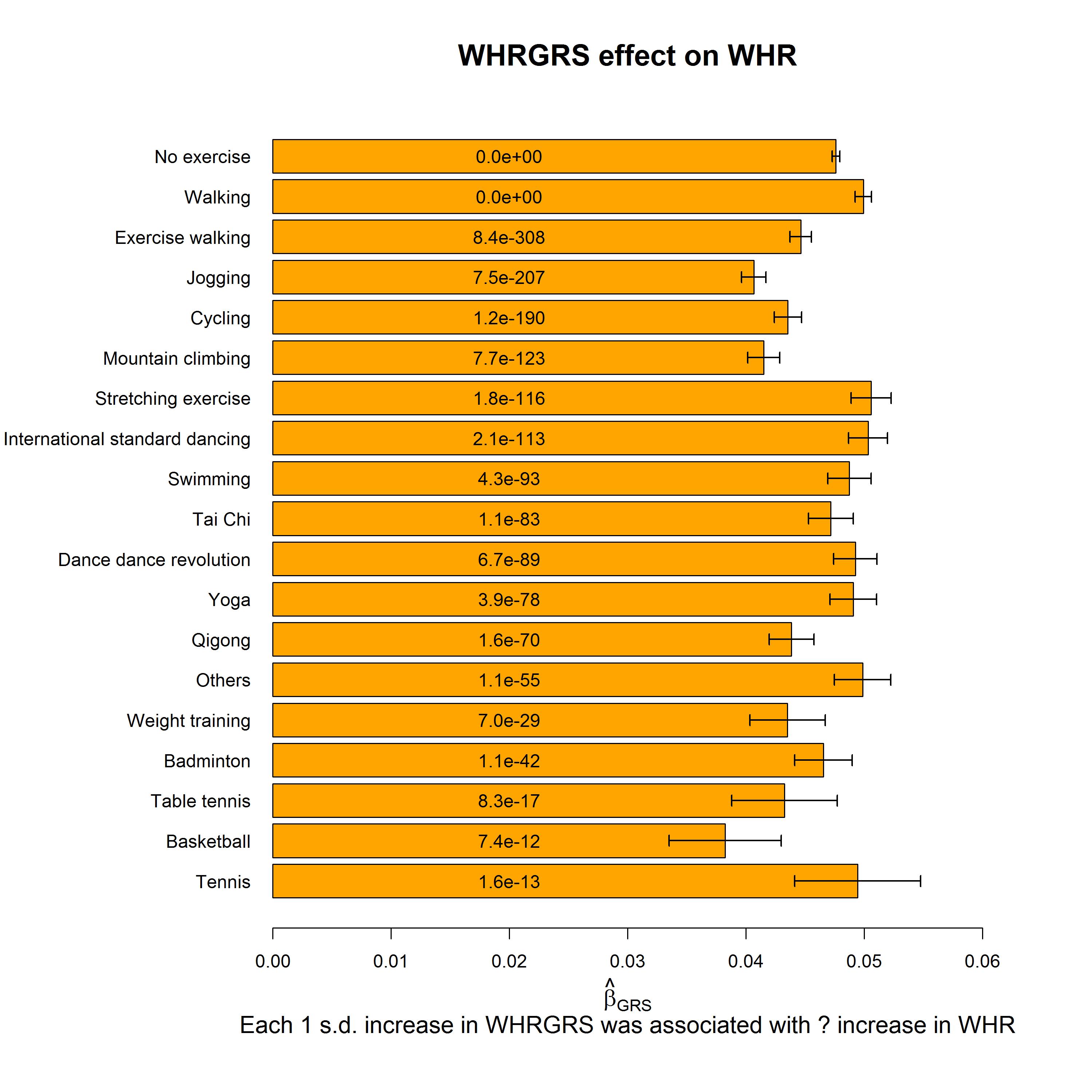

Supplement: S4 Fig — The regression model (stratified by exercise types) was built as WHR = β0 + βGRSWHRGRS + βCCovariates + ε, where WHRGRS was calculated at the marginal-association P-value threshold of 0.05. We used this WHRGRS for plots because 0.05 is generally considered as the significance level in statistical analyses. The orange bars represent β^GRS on WHR (stratified by exercise types), and the black segments mark [β^GRS−standarderrorofβ^GRS,β^GRS+standarderrorofβ^GRS]. The text on each bar is the P-value of testing H0: βGRS = 0 vs. H1: βGRS ≠ 0. Covariates adjusted in the regression model included sex, age, educational attainment, drinking status, smoking status, and the first 10 PCs. Consistent with Table 3, the 18 kinds of exercise were sorted according to popularity. (JPG) [file pgen.1008277.s004.jpg]
