# Supplementary material for: Performing different kinds of physical exercise differentially attenuates the genetic effects on obesity measures: Evidence from 18,424 Taiwan Biobank participants
Source: PLoS Genet. 2019 Aug 1;15(8):e1008277. doi: 10.1371/journal.pgen.1008277 (PMC6675047; doi:10.1371/journal.pgen.1008277)
Supplement: S2 Table — (DOCX) [file pgen.1008277.s006.docx]

|  | ${\hat{\boldsymbol{\beta}}}_{\boldsymbol{EuGRS}}$ | Std. Error | Test statistic | *P*-value |
| --- | --- | --- | --- | --- |
| BMI (kg/m^2^) | 0.411974 ^1^ | 0.025828 | 15.95056 | 6.8E-57 |
| Body fat % | 0.529964 ^2^ | 0.044212 | 11.98675 | 5.6E-33 |
| Waist circumference (cm) | 0.914484 ^3^ | 0.067834 | 13.48112 | 3.2E-41 |
| Hip circumference (cm) | 0.659362 ^4^ | 0.049500 | 13.32040 | 2.7E-40 |
| Waist-to-hip ratio | 0.003455 ^5^ | 0.000433 | 7.982458 | 1.5E-15 |

**S2 Table.** The association of European-based GRS with the 5 obesity measures

1. The regression model was built as $BMI=\beta_{0}+\beta_{EuGRS}EuGRS+\boldsymbol{\beta}_{\boldsymbol{C}}\boldsymbol{Covariates}+\varepsilon$. Covariates adjusted in the regression models included sex, age, educational attainment, drinking status, smoking status, and the first 10 PCs. ${\hat{\boldsymbol{\beta}}}_{EuGRS}=0.41$ indicates that each 1 s.d. increase in EuGRS was associated with a 0.41 kg/m^2^ increase in BMI ($p=6.8\times{10}^{-57}$).
2. The regression model was built as $BFP=\beta_{0}+\beta_{EuGRS}EuGRS+\boldsymbol{\beta}_{\boldsymbol{C}}\boldsymbol{Covariates}+\varepsilon$. Covariates adjusted in the regression models included sex, age, educational attainment, drinking status, smoking status, and the first 10 PCs. ${\hat{\boldsymbol{\beta}}}_{EuGRS}=0.53$ indicates that each 1 s.d. increase in EuGRS was associated with a 0.53 % increase in BFP ($p=5.6\times{10}^{-33}$).
3. The regression model was built as *WC*$=\beta_{0}+\beta_{EuGRS}EuGRS+\boldsymbol{\beta}_{\boldsymbol{C}}\boldsymbol{Covariates}+\varepsilon$. Covariates adjusted in the regression models included sex, age, educational attainment, drinking status, smoking status, and the first 10 PCs. ${\hat{\boldsymbol{\beta}}}_{EuGRS}=0.91$ indicates that each 1 s.d. increase in EuGRS was associated with a 0.91 cm increase in WC ($p=3.2\times{10}^{-41}$).
4. The regression model was built as $HC=\beta_{0}+\beta_{EuGRS}EuGRS+\boldsymbol{\beta}_{\boldsymbol{C}}\boldsymbol{Covariates}+\varepsilon$. Covariates adjusted in the regression models included sex, age, educational attainment, drinking status, smoking status, and the first 10 PCs. ${\hat{\boldsymbol{\beta}}}_{EuGRS}=0.66$ indicates that each 1 s.d. increase in EuGRS was associated with a 0.66 cm increase in HC ($p=2.7\times{10}^{-40}$).
5. The regression model was built as $WHR=\beta_{0}+\beta_{EuGRS}EuGRS+\boldsymbol{\beta}_{\boldsymbol{C}}\boldsymbol{Covariates}+\varepsilon$. Covariates adjusted in the regression models included sex, age, educational attainment, drinking status, smoking status, and the first 10 PCs. ${\hat{\boldsymbol{\beta}}}_{EuGRS}=0.003$ indicates that each 1 s.d. increase in EuGRS was associated with a 0.003 increase in WHR ($p=1.5\times{10}^{-15}$).
