# Supplementary material for: Performing different kinds of physical exercise differentially attenuates the genetic effects on obesity measures: Evidence from 18,424 Taiwan Biobank participants
Source: PLoS Genet. 2019 Aug 1;15(8):e1008277. doi: 10.1371/journal.pgen.1008277 (PMC6675047; doi:10.1371/journal.pgen.1008277)
Supplement: S3 Table — (DOCX) [file pgen.1008277.s007.docx]

|  | | | | BMI (kg/m^2^) | | Body fat % | | Waist circumference (cm) | | Hip circumference (cm) | | Waist-to-hip ratio | |
| --- | --- | --- | --- | --- | --- | --- | --- | --- | --- | --- | --- | --- | --- |
|  | **No. of subjects** | **% of males** | **Age (years), mean (s.d.)** | ${\hat{\boldsymbol{\beta}}}_{\boldsymbol{Int}}$ | ***P*-value** | ${\hat{\boldsymbol{\beta}}}_{\boldsymbol{Int}}$ | ***P*-value** | ${\hat{\boldsymbol{\beta}}}_{\boldsymbol{Int}}$ | ***P*-value** | ${\hat{\boldsymbol{\beta}}}_{\boldsymbol{Int}}$ | ***P*-value** | ${\hat{\boldsymbol{\beta}}}_{\boldsymbol{Int}}$ | ***P*-value** |
| Regular exercise | 7,652 | 50.9 | 53.5 (10.3) | -0.15 | 0.0047 | -0.18 | 0.0386 | -0.36 | 0.0094 | -0.19 | 0.0556 | -0.0021 | 0.0163 |
| Specific analysis for kinds of exercise: Some subjects engage in 2 or 3 kinds of regular exercise.  The following 18 kinds of exercise were sorted according to popularity. | | | | | | | | | | | | | |
| Walking | 2,637 | 47.3 | 55.8 (9.2) | -0.035 | 0.634 | -0.078 | 0.538 | -0.127 | 0.513 | 0.020 | 0.887 | -0.00165 | 0.183 |
| Exercise walking | 1,439 | 52.3 | 54.6 (9.3) | -0.043 | 0.660 | -0.014 | 0.931 | -0.181 | 0.476 | -0.013 | 0.945 | -0.00162 | 0.315 |
| Jogging | 1,107 | 81.1 | 45.4 (10.1) | -0.179 | 0.108 | -0.317 | 0.092 | -0.514 | 0.078 | -0.533 | 0.012 | -0.00072 | 0.697 |
| Cycling | 989 | 68.6 | 51.4 (10.4) | -0.207 | 0.074 | -0.263 | 0.186 | -0.304 | 0.318 | -0.129 | 0.562 | -0.00206 | 0.290 |
| Mountain climbing | 628 | 57.3 | 55.2 (8.2) | -0.092 | 0.535 | -0.148 | 0.556 | -0.607 | 0.116 | -0.312 | 0.270 | -0.00342 | 0.166 |
| Stretching exercise | 602 | 33.9 | 58.1 (8.4) | -0.034 | 0.809 | -0.076 | 0.753 | -0.057 | 0.878 | -0.078 | 0.773 | -0.00011 | 0.963 |
| International standard dancing | 513 | 13.8 | 56.8 (7.7) | -0.169 | 0.289 | -0.104 | 0.705 | -0.064 | 0.878 | -0.151 | 0.620 | 0.00093 | 0.729 |
| Swimming | 486 | 66.5 | 52.7 (10.7) | 0.056 | 0.727 | -0.051 | 0.854 | -0.128 | 0.763 | 0.268 | 0.386 | -0.00372 | 0.168 |
| Tai Chi | 449 | 55.7 | 56.5 (9.1) | -0.054 | 0.745 | 0.084 | 0.767 | -0.388 | 0.374 | -0.446 | 0.162 | 0.00016 | 0.955 |
| Dance dance revolution | 420 | 8.3 | 50.5 (10.6) | -0.267 | 0.128 | -0.324 | 0.278 | -0.682 | 0.139 | -0.606 | 0.072 | -0.00120 | 0.684 |
| Yoga | 379 | 10.3 | 51.5 (9.8) | -0.068 | 0.723 | 0.129 | 0.695 | -0.062 | 0.902 | 0.068 | 0.853 | -0.00110 | 0.731 |
| Qigong | 377 | 36.3 | 58.1 (7.8) | -0.276 | 0.122 | -0.480 | 0.117 | -0.366 | 0.433 | -0.395 | 0.247 | -0.00046 | 0.878 |
| Others | 285 | 41.4 | 53.5 (11.7) | -0.062 | 0.742 | -0.258 | 0.425 | -0.238 | 0.633 | 0.168 | 0.643 | -0.00441 | 0.165 |
| Weight training | 218 | 72.9 | 45.4 (11.3) | 0.049 | 0.848 | 0.333 | 0.445 | 0.688 | 0.306 | 0.002 | 0.996 | 0.00663 | 0.122 |
| Badminton | 204 | 78.9 | 46.0 (9.5) | -0.019 | 0.945 | 0.149 | 0.744 | 0.758 | 0.286 | -0.036 | 0.945 | 0.00872 | 0.055 |
| Table tennis | 169 | 76.3 | 54.1 (10.6) | -0.280 | 0.299 | -0.478 | 0.294 | -0.568 | 0.422 | -0.581 | 0.260 | -0.00039 | 0.930 |
| Basketball | 119 | 97.5 | 40.8 (9.0) | -0.216 | 0.500 | -0.202 | 0.715 | -0.584 | 0.487 | -0.631 | 0.304 | -0.00081 | 0.880 |
| Tennis | 110 | 80.9 | 54.2 (10.0) | 0.403 | 0.226 | 0.909 | 0.141 | 0.912 | 0.295 | 0.553 | 0.385 | 0.00393 | 0.480 |

**S3 Table.** Interaction between EuGRS and exercise on each obesity measure
