# Supplementary material for: Performing different kinds of physical exercise differentially attenuates the genetic effects on obesity measures: Evidence from 18,424 Taiwan Biobank participants
Source: PLoS Genet. 2019 Aug 1;15(8):e1008277. doi: 10.1371/journal.pgen.1008277 (PMC6675047; doi:10.1371/journal.pgen.1008277)
Supplement: S4 Table — (DOCX) [file pgen.1008277.s008.docx]

|  | Frequency per month | | BMI (kg/m^2^) | | Body fat % | | Waist circumference (cm) | | Hip circumference (cm) | | Waist-to-hip ratio | |
| --- | --- | --- | --- | --- | --- | --- | --- | --- | --- | --- | --- | --- |
|  | **Mean** | **Standard deviation** | ${\hat{\boldsymbol{\beta}}}_{\boldsymbol{Int}}$ | ***P*-value** | ${\hat{\boldsymbol{\beta}}}_{\boldsymbol{Int}}$ | ***P*-value** | ${\hat{\boldsymbol{\beta}}}_{\boldsymbol{Int}}$ | ***P*-value** | ${\hat{\boldsymbol{\beta}}}_{\boldsymbol{Int}}$ | ***P*-value** | ${\hat{\boldsymbol{\beta}}}_{\boldsymbol{Int}}$ | ***P*-value** |
| Walking | 20.2 | 9.6 | -0.0004 | 0.901 | -0.0002 | 0.977 | -0.0027 | 0.751 | 0.0016 | 0.789 | -0.000046 | 0.393 |
| Exercise walking | 18.5 | 8.8 | -0.0035 | 0.461 | -0.0010 | 0.898 | -0.0072 | 0.560 | -0.0046 | 0.612 | -0.000022 | 0.779 |
| Jogging | 14.5 | 7.7 | -0.0089 | 0.152 | -0.0152 | 0.146 | -0.0223 | 0.172 | -0.0238 | 0.046 | -0.000021 | 0.840 |
| Cycling | 15.2 | 9.8 | -0.0125 | 0.057 | -0.0175 | 0.121 | -0.0252 | 0.143 | -0.0222 | 0.077 | -0.000056 | 0.612 |
| Mountain climbing | 9.0 | 7.9 | -0.0073 | 0.563 | -0.0173 | 0.426 | -0.0247 | 0.456 | -0.0208 | 0.392 | -0.000071 | 0.738 |
| Stretching exercise | 21.3 | 8.4 | -0.0014 | 0.825 | -0.0042 | 0.697 | -0.0030 | 0.859 | -0.0009 | 0.941 | -0.000033 | 0.760 |
| International standard dancing | 16.3 | 8.2 | -0.0078 | 0.343 | -0.0093 | 0.509 | -0.0039 | 0.858 | -0.0077 | 0.625 | 0.000037 | 0.789 |
| Swimming | 15.4 | 9.6 | 0.0019 | 0.821 | -0.0083 | 0.576 | -0.0029 | 0.897 | 0.0153 | 0.351 | -0.000168 | 0.241 |
| Tai Chi | 18.7 | 9.5 | 0.0004 | 0.958 | 0.0098 | 0.471 | -0.0140 | 0.502 | -0.0176 | 0.245 | 0.000022 | 0.871 |
| Dance dance revolution | 14.1 | 7.7 | -0.0213 | 0.043 | -0.0279 | 0.118 | -0.0445 | 0.107 | -0.0425 | 0.035 | -0.000057 | 0.747 |
| Yoga | 11.9 | 8.1 | -0.0091 | 0.465 | 0.0043 | 0.840 | -0.0167 | 0.609 | -0.0083 | 0.729 | -0.000075 | 0.720 |
| Qigong | 21.4 | 9.4 | -0.0082 | 0.263 | -0.0179 | 0.161 | -0.0073 | 0.704 | -0.0128 | 0.362 | 0.000033 | 0.789 |
| Others | 18.3 | 11.9 | -0.0053 | 0.536 | -0.0108 | 0.459 | -0.0147 | 0.514 | -0.0014 | 0.934 | -0.000162 | 0.260 |
| Weight training | 15.4 | 9.1 | -0.0054 | 0.714 | 0.0138 | 0.580 | 0.0250 | 0.520 | -0.0134 | 0.635 | 0.000361 | 0.146 |
| Badminton | 11.4 | 6.9 | -0.0045 | 0.837 | -0.0038 | 0.918 | 0.0332 | 0.563 | -0.0069 | 0.870 | 0.000436 | 0.235 |
| Table tennis | 15.7 | 8.4 | -0.0136 | 0.370 | -0.0258 | 0.313 | -0.0485 | 0.222 | -0.0342 | 0.237 | -0.000180 | 0.478 |
| Basketball | 10.9 | 7.6 | -0.0397 | 0.113 | -0.0463 | 0.295 | -0.0961 | 0.143 | -0.0886 | 0.064 | -0.000227 | 0.587 |
| Tennis | 16.7 | 8.4 | 0.0170 | 0.325 | 0.0321 | 0.293 | 0.0159 | 0.727 | 0.0206 | 0.534 | -0.000051 | 0.860 |

**S4 Table.** Interaction between EuGRS and exercise frequency per month
