# Supplementary material for: Performing different kinds of physical exercise differentially attenuates the genetic effects on obesity measures: Evidence from 18,424 Taiwan Biobank participants
Source: PLoS Genet. 2019 Aug 1;15(8):e1008277. doi: 10.1371/journal.pgen.1008277 (PMC6675047; doi:10.1371/journal.pgen.1008277)
Supplement: S5 Table — (DOCX) [file pgen.1008277.s009.docx]

|  | Duration for each exercise (hours) | | BMI (kg/m^2^) | | Body fat % | | Waist circumference (cm) | | Hip circumference (cm) | | Waist-to-hip ratio | |
| --- | --- | --- | --- | --- | --- | --- | --- | --- | --- | --- | --- | --- |
|  | **Mean** | **Standard deviation** | ${\hat{\boldsymbol{\beta}}}_{\boldsymbol{Int}}$ | ***P*-value** | ${\hat{\boldsymbol{\beta}}}_{\boldsymbol{Int}}$ | ***P*-value** | ${\hat{\boldsymbol{\beta}}}_{\boldsymbol{Int}}$ | ***P*-value** | ${\hat{\boldsymbol{\beta}}}_{\boldsymbol{Int}}$ | ***P*-value** | ${\hat{\boldsymbol{\beta}}}_{\boldsymbol{Int}}$ | ***P*-value** |
| Walking | 0.78 | 0.39 | -0.069 | 0.410 | -0.090 | 0.530 | -0.166 | 0.450 | -0.017 | 0.917 | -0.00181 | 0.198 |
| Exercise walking | 0.81 | 0.38 | -0.099 | 0.369 | -0.097 | 0.608 | -0.376 | 0.195 | -0.070 | 0.740 | -0.00320 | 0.084 |
| Jogging | 0.70 | 0.33 | -0.268 | 0.066 | -0.426 | 0.083 | -0.838 | 0.028 | -0.896 | 0.001 | -0.00078 | 0.749 |
| Cycling | 1.16 | 0.89 | -0.032 | 0.670 | -0.035 | 0.785 | 0.188 | 0.343 | 0.130 | 0.367 | 0.00061 | 0.630 |
| Mountain climbing | 1.99 | 1.22 | -0.061 | 0.388 | -0.071 | 0.554 | -0.338 | 0.067 | -0.213 | 0.114 | -0.00150 | 0.203 |
| Stretching exercise | 0.73 | 0.36 | 0.105 | 0.559 | 0.149 | 0.624 | 0.507 | 0.280 | 0.245 | 0.476 | 0.00295 | 0.326 |
| International standard dancing | 1.28 | 0.61 | -0.060 | 0.599 | -0.009 | 0.963 | 0.038 | 0.899 | -0.023 | 0.914 | 0.00075 | 0.691 |
| Swimming | 0.84 | 0.50 | -0.074 | 0.670 | -0.156 | 0.614 | -0.431 | 0.345 | 0.045 | 0.892 | -0.00474 | 0.104 |
| Tai Chi | 1.13 | 0.54 | 0.075 | 0.593 | 0.230 | 0.339 | 0.053 | 0.885 | -0.100 | 0.708 | 0.00157 | 0.503 |
| Dance dance revolution | 1.01 | 0.43 | -0.168 | 0.308 | -0.204 | 0.467 | -0.564 | 0.192 | -0.561 | 0.076 | -0.00039 | 0.887 |
| Yoga | 1.17 | 0.49 | -0.141 | 0.362 | -0.129 | 0.633 | -0.270 | 0.506 | -0.081 | 0.784 | -0.00200 | 0.439 |
| Qigong | 1.01 | 0.46 | -0.112 | 0.454 | -0.240 | 0.343 | -0.098 | 0.802 | -0.160 | 0.576 | 0.00027 | 0.913 |
| Others | 0.96 | 0.65 | 0.039 | 0.800 | -0.101 | 0.696 | 0.012 | 0.976 | 0.227 | 0.439 | -0.00216 | 0.400 |
| Weight training | 0.80 | 0.48 | 0.245 | 0.410 | 0.452 | 0.369 | 1.075 | 0.168 | 0.447 | 0.433 | 0.00662 | 0.183 |
| Badminton | 1.40 | 0.60 | -0.037 | 0.827 | -0.002 | 0.994 | 0.318 | 0.477 | -0.064 | 0.844 | 0.00418 | 0.143 |
| Table tennis | 1.34 | 0.59 | -0.291 | 0.137 | -0.440 | 0.181 | -0.645 | 0.208 | -0.596 | 0.112 | -0.00106 | 0.745 |
| Basketball | 1.40 | 0.68 | -0.111 | 0.592 | -0.080 | 0.823 | -0.217 | 0.689 | -0.203 | 0.609 | -0.00058 | 0.867 |
| Tennis | 1.41 | 0.64 | 0.288 | 0.204 | 0.589 | 0.198 | 0.738 | 0.214 | 0.445 | 0.305 | 0.00332 | 0.382 |

**S5 Table.** Interaction between EuGRS and exercise duration (in hours)
