# Supplementary material for: Performing different kinds of physical exercise differentially attenuates the genetic effects on obesity measures: Evidence from 18,424 Taiwan Biobank participants
Source: PLoS Genet. 2019 Aug 1;15(8):e1008277. doi: 10.1371/journal.pgen.1008277 (PMC6675047; doi:10.1371/journal.pgen.1008277)
Supplement: S6 Table — (DOCX) [file pgen.1008277.s010.docx]

|  | Cumulative variance explained by the 86 SNPs |
| --- | --- |
| BMI (kg/m^2^) | 1.92 % ^1^ |
| Body fat % | 1.05 % ^2^ |
| Waist circumference (cm) | 1.43 % ^3^ |
| Hip circumference (cm) | 1.60 % ^4^ |
| Waist-to-hip ratio | 0.79 % ^5^ |

**S6 Table.** The cumulative variance explained by the 86 European BMI-associated SNPs

1. The R-square of the regression model $BMI=\beta_{0}+\beta_{SNP,1}{SNP}_{1}+\cdots+\beta_{SNP,86}{SNP}_{86}+\varepsilon$ was 1.92%.
2. The R-square of the regression model $BFP=\beta_{0}+\beta_{SNP,1}{SNP}_{1}+\cdots+\beta_{SNP,86}{SNP}_{86}+\varepsilon$ was 1.05%.
3. The R-square of the regression model $WC=\beta_{0}+\beta_{SNP,1}{SNP}_{1}+\cdots+\beta_{SNP,86}{SNP}_{86}+\varepsilon$ was 1.43%.
4. The R-square of the regression model $HC=\beta_{0}+\beta_{SNP,1}{SNP}_{1}+\cdots+\beta_{SNP,86}{SNP}_{86}+\varepsilon$ was 1.60%.
5. The R-square of the regression model $WHR=\beta_{0}+\beta_{SNP,1}{SNP}_{1}+\cdots+\beta_{SNP,86}{SNP}_{86}+\varepsilon$ was 0.79%.
