# Supplementary material for: Performing different kinds of physical exercise differentially attenuates the genetic effects on obesity measures: Evidence from 18,424 Taiwan Biobank participants
Source: PLoS Genet. 2019 Aug 1;15(8):e1008277. doi: 10.1371/journal.pgen.1008277 (PMC6675047; doi:10.1371/journal.pgen.1008277)
Supplement: S7 Table — (DOCX) [file pgen.1008277.s011.docx]

| *P*-value threshold | 0.0001 | 0.00025 | 0.0005 | 0.001 | 0.0025 | 0.005 | 0.01 | 0.025 | 0.05 | 0.1 |
| --- | --- | --- | --- | --- | --- | --- | --- | --- | --- | --- |
| BMI | 24 | 66 | 116 | 209 | **481** | 870 | 1,690 | **4,047** | **7,753** | 15,206 |
| R-square explained by BMIGRS ^1^ | 2.1% | 4.8% | 7.4% | 11.5% | 20.2% | 28.3% | 38.6% | 52.6% | 61.1% | 68.2% |
| Number of times a significant BMIGRS-exercise interaction was detected at this marginal-association *P*-value threshold | | | | | **2** |  |  | **7** | **7** |  |
| Body fat % | 28 | 54 | 106 | 195 | 447 | **865** | **1,638** | **4,101** | 7,890 | 15,182 |
| R-square explained by BFPGRS ^2^ | 1.4% | 2.7% | 4.6% | 7.2% | 13.2% | 19.9% | 26.9% | 37.3% | 43.5% | 48.0% |
| Number of times a significant BFPGRS-exercise interaction was detected at this marginal-association *P*-value threshold | | | | |  | **1** ^3^ | **1** | **2** |  |  |
| Waist circumference | 14 | 47 | 96 | 181 | 444 | 830 | 1,621 | **3,987** | 7,772 | 15,023 |
| R-square explained by WCGRS ^4^ | 1.1% | 3.1% | 5.7% | 9.0% | 17.3% | 25.0% | 34.9% | 48.2% | 56.1% | 62.5% |
| Number of times a significant WCGRS-exercise interaction was detected at this marginal-association *P*-value threshold | | | | |  |  |  | **1** |  |  |
| Hip circumference | 24 | 54 | 110 | 214 | 487 | 862 | **1,652** | 3,981 | 7,741 | 15,161 |
| R-square explained by HCGRS ^5^ | 2.1% | 4.2% | 7.4% | 12.0% | 20.8% | 28.9% | 39.7% | 54.2% | 63.3% | 70.4% |
| Number of times a significant HCGRS-exercise interaction was detected at this marginal-association *P*-value threshold | | | | |  |  | **5** |  |  |  |
| Waist-to-hip ratio | 24 | 58 | 91 | 196 | 449 | 864 | 1,599 | 3,926 | 7,678 | 14,990 |
| R-square explained by WHRGRS ^6^ | 1.6% | 3.5% | 4.9% | 8.7% | 15.8% | 23.1% | 30.7% | 42.1% | 49.7% | 55.7% |

**S7 Table.** The numbers of SNPs used to form the GRSs under 10 *P*-value thresholds

^1^ R-square explained by BMIGRS: this is the R-square of the model BMI = $\beta_{0}$+$\beta_{GRS}$BMIGRS + $\varepsilon$.

^2^ R-square explained by BFPGRS: this is the R-square of the model BFP = $\beta_{0}$+$\beta_{GRS}$BFPGRS + $\varepsilon$.

^3^ The significant interaction between BFPGRS and regular exercise was detected at the marginal-association *P*-value threshold of 0.005, where 865 SNPs were used to construct the BFPGRS (shown in the first row of Table 3).

^4^ R-square explained by WCGRS: this is the R-square of the model WC = $\beta_{0}$+$\beta_{GRS}$WCGRS + $\varepsilon$.

^5^ R-square explained by HCGRS: this is the R-square of the model HC = $\beta_{0}$+$\beta_{GRS}$HCGRS + $\varepsilon$.

^6^ R-square explained by WHRGRS: this is the R-square of the model WHR = $\beta_{0}$+$\beta_{GRS}$WHRGRS + $\varepsilon$.
