# Supplementary material for: Performing different kinds of physical exercise differentially attenuates the genetic effects on obesity measures: Evidence from 18,424 Taiwan Biobank participants
Source: PLoS Genet. 2019 Aug 1;15(8):e1008277. doi: 10.1371/journal.pgen.1008277 (PMC6675047; doi:10.1371/journal.pgen.1008277)
Supplement: S8 Table — (DOCX) [file pgen.1008277.s012.docx]

| Regular exercise x 5 obesity measures = 5 tests  18 kinds of exercise x 5 obesity measures = 90 tests | | | | BMI (kg/m^2^) | | Body fat % | | Waist circumference (cm) | | Hip circumference (cm) | | Waist-to-hip ratio | |
| --- | --- | --- | --- | --- | --- | --- | --- | --- | --- | --- | --- | --- | --- |
|  | **No. of subjects** | **% of males** | **Age (years), mean (s.d.)** | ${\hat{\boldsymbol{\beta}}}_{\boldsymbol{E}}$ | ***P*-value** | ${\hat{\boldsymbol{\beta}}}_{\boldsymbol{E}}$ | ***P*-value** | ${\hat{\boldsymbol{\beta}}}_{\boldsymbol{E}}$ | ***P*-value** | ${\hat{\boldsymbol{\beta}}}_{\boldsymbol{E}}$ | ***P*-value** | ${\hat{\boldsymbol{\beta}}}_{\boldsymbol{E}}$ | ***P*-value** |
| Regular exercise | 7 652 | 50.9 | 53.5 (10.3) | -0.13 ^1^ | 4.9E-04 | **-0.57** | **2.0E-12** | **-0.62** | **1.7E-10** | **-0.42** | **4.9E-07** | **-0.006** | **8.8E-11** |
| Specific analysis for kinds of exercise: Some subjects engage in 2 or 3 kinds of regular exercise.  The following 18 kinds of exercise were sorted according to popularity. | | | | | | | | | | | | | |
| Walking | 2 637 | 47.3 | 55.8 (9.2) | 0.00 | 9.8E-01 | 0.06 | 4.6E-01 | 0.05 | 7.2E-01 | 0.02 | 8.4E-01 | 0.00116 | 2.8E-01 |
| Exercise walking | 1 439 | 52.3 | 54.6 (9.3) | 0.01 | 8.3E-01 | -0.11 | 3.8E-01 | -0.30 | 2.0E-01 | -0.01 | 9.6E-01 | -0.00077 | 6.0E-01 |
| Jogging | 1 107 | 81.1 | 45.4 (10.1) | -0.23 | 6.2E-04 | **-0.56** ^2^ | **9.1E-06** | **-0.65** | **2.5E-05** | -0.57 | 6.3E-04 | **-0.00482** | **1.5E-05** |
| Cycling | 989 | 68.6 | 51.4 (10.4) | 0.20 | 8.0E-02 | 0.04 | 8.2E-01 | -0.12 | 5.5E-01 | 0.13 | 5.3E-01 | -0.00380 | 3.8E-02 |
| Mountain climbing | 628 | 57.3 | 55.2 (8.2) | -0.13 | 1.8E-01 | -0.31 | 1.4E-02 | -0.31 | 1.1E-01 | -0.17 | 2.4E-01 | -0.00252 | 1.8E-01 |
| Stretching exercise | 602 | 33.9 | 58.1 (8.4) | -0.25 | 2.8E-02 | -0.64 | 1.1E-02 | -0.34 | 2.6E-01 | -0.21 | 3.4E-01 | 0.00034 | 8.7E-01 |
| International standard dancing | 513 | 13.8 | 56.8 (7.7) | -0.02 | 8.4E-01 | -0.14 | 3.6E-01 | -0.35 | 1.0E-01 | 0.05 | 7.5E-01 | -0.00131 | 3.4E-01 |
| Swimming | 486 | 66.5 | 52.7 (10.7) | -0.09 | 5.2E-01 | -0.49 | 5.0E-02 | -0.52 | 3.9E-02 | -0.14 | 3.8E-01 | -0.00547 | 3.3E-02 |
| Tai Chi | 449 | 55.7 | 56.5 (9.1) | -0.45 | 1.6E-03 | **-1.07** | **3.2E-05** | **-1.74** | **3.6E-07** | -0.93 | 1.9E-04 | -0.00801 | 2.6E-03 |
| Dance dance revolution | 420 | 8.3 | 50.5 (10.6) | -0.12 | 2.5E-01 | -0.34 | 1.7E-01 | -0.73 | 1.8E-02 | -0.34 | 9.0E-02 | -0.00055 | 7.2E-01 |
| Yoga | 379 | 10.3 | 51.5 (9.8) | **-0.73** | **1.6E-05** | -0.24 | 1.6E-01 | **-2.17** | **1.0E-06** | **-1.46** | **1.5E-05** | -0.00555 | 5.8E-04 |
| Qigong | 377 | 36.3 | 58.1 (7.8) | -0.30 | 9.7E-02 | -0.46 | 3.7E-03 | -0.27 | 5.0E-01 | -0.19 | 5.6E-01 | 0.00067 | 7.9E-01 |
| Others | 285 | 41.4 | 53.5 (11.7) | 0.00 | 9.9E-01 | 0.34 | 2.9E-01 | 0.15 | 7.9E-01 | -0.31 | 3.2E-01 | 0.00167 | 5.9E-01 |
| Weight training | 218 | 72.9 | 45.4 (11.3) | -0.01 | 9.5E-01 | -0.22 | 2.8E-01 | 0.13 | 7.3E-01 | 0.11 | 6.6E-01 | -0.00367 | 3.5E-01 |
| Badminton | 204 | 78.9 | 46.0 (9.5) | 0.21 | 4.0E-01 | 0.24 | 4.5E-01 | -0.08 | 8.2E-01 | 0.84 | 7.2E-02 | -0.01066 | 8.1E-03 |
| Table tennis | 169 | 76.3 | 54.1 (10.6) | -0.50 | 1.6E-02 | -0.51 | 5.9E-02 | -1.15 | 7.0E-03 | -0.43 | 1.1E-01 | **-0.01595** | **9.0E-05** |
| Basketball | 119 | 97.5 | 40.8 (9.0) | 0.09 | 6.8E-01 | 0.09 | 8.8E-01 | -0.17 | 7.4E-01 | 0.74 | 2.3E-01 | -0.00432 | 1.2E-01 |
| Tennis | 110 | 80.9 | 54.2 (10.0) | 0.06 | 8.6E-01 | -0.31 | 5.7E-01 | -1.08 | 2.2E-01 | 0.13 | 8.4E-01 | -0.00204 | 4.8E-01 |

**S8 Table.** Main associations of exercises with obesity measures (significant results with *p* < 9.1x10^-5^ are highlighted)

1. Performing regular physical exercise was associated with a 0.13 kg/m^2^ decrease in BMI. The regression model was built as BMI = $\beta_{0}$+$\beta_{GRS}$BMIGRS +$\beta_{E}$Regular exercise +$\beta_{Int}$BMIGRS x Regular exercise +$\boldsymbol{\beta}_{C}$**Covariates** + $\varepsilon$. Covariates adjusted in the regression model included sex, age, educational attainment, drinking status, smoking status, and the first 10 PCs.
2. Jogging was associated with a 0.56% decrease in BFP. The regression model was built as BFP = $\beta_{0}$+$\beta_{GRS}$BFPGRS + $\beta_{E}$Regular jogging + $\beta_{Int}$BFPGRS x Regular jogging + $\boldsymbol{\beta}_{C}$**Covariates** + $\varepsilon$. Covariates adjusted in the regression model included sex, age, educational attainment, drinking status, smoking status, the first 10 PCs, 17 covariates regarding engaging in the other 17 kinds of exercise or not, and the interaction terms between BFPGRS and the 17 kinds of exercise.
