# Supplementary material for: Performing different kinds of physical exercise differentially attenuates the genetic effects on obesity measures: Evidence from 18,424 Taiwan Biobank participants
Source: PLoS Genet. 2019 Aug 1;15(8):e1008277. doi: 10.1371/journal.pgen.1008277 (PMC6675047; doi:10.1371/journal.pgen.1008277)
Supplement: S9 Table — (DOCX) [file pgen.1008277.s013.docx]

|  | Frequency per month | | BMI (kg/m^2^) | | Body fat % | | Waist circumference (cm) | | Hip circumference (cm) | | Waist-to-hip ratio | |
| --- | --- | --- | --- | --- | --- | --- | --- | --- | --- | --- | --- | --- |
|  | **Mean** | **Standard deviation** | ${\hat{\boldsymbol{\beta}}}_{\boldsymbol{E}}$ | ***P*-value** | ${\hat{\boldsymbol{\beta}}}_{\boldsymbol{E}}$ | ***P*-value** | ${\hat{\boldsymbol{\beta}}}_{\boldsymbol{E}}$ | ***P*-value** | ${\hat{\boldsymbol{\beta}}}_{\boldsymbol{E}}$ | ***P*-value** | ${\hat{\boldsymbol{\beta}}}_{\boldsymbol{E}}$ | ***P*-value** |
| Walking | 20.2 | 9.6 | 0.000 | 9.5E-01 | 0.002 | 5.9E-01 | 0.002 | 7.0E-01 | 0.002 | 6.6E-01 | 0.00005 | 2.7E-01 |
| Exercise walking | 18.5 | 8.8 | -0.005 | 2.4E-01 | -0.012 | 5.3E-02 | -0.022 | 5.0E-02 | -0.006 | 3.9E-01 | -0.00006 | 2.4E-01 |
| Jogging | 14.5 | 7.7 | **-0.016 ^1^** | **3.3E-05** | **-0.039** | **2.0E-07** | **-0.072** | **1.7E-07** | **-0.039** | **6.8E-05** | **-0.00045** | **2.3E-07** |
| Cycling | 15.2 | 9.8 | -0.001 | 9.4E-01 | -0.012 | 1.8E-01 | -0.031 | 4.9E-02 | -0.005 | 6.2E-01 | -0.00024 | 1.6E-02 |
| Mountain climbing | 9.0 | 7.9 | -0.009 | 2.6E-01 | -0.027 | 1.0E-02 | -0.038 | 3.6E-02 | -0.010 | 4.2E-01 | -0.00022 | 1.6E-01 |
| Stretching exercise | 21.3 | 8.4 | -0.013 | 3.4E-02 | -0.033 | 2.4E-03 | -0.012 | 2.8E-01 | -0.021 | 7.6E-02 | 0.00003 | 7.2E-01 |
| International standard dancing | 16.3 | 8.2 | -0.003 | 6.2E-01 | -0.013 | 1.4E-01 | -0.025 | 3.3E-02 | 0.000 | 1 | -0.00028 | 1.9E-02 |
| Swimming | 15.4 | 9.6 | 0.001 | 8.7E-01 | -0.014 | 3.3E-01 | -0.014 | 2.5E-01 | -0.033 | 2.9E-02 | -0.00026 | 5.8E-02 |
| Tai Chi | 18.7 | 9.5 | -0.017 | 2.8E-02 | **-0.052** | **2.6E-05** | **-0.073** | **6.4E-06** | -0.038 | 1.4E-03 | -0.00033 | 9.6E-03 |
| Dance dance revolution | 14.1 | 7.7 | -0.007 | 2.9E-01 | -0.039 | 1.8E-02 | -0.044 | 2.0E-02 | -0.021 | 8.6E-02 | -0.00006 | 5.5E-01 |
| Yoga | 11.9 | 8.1 | **-0.063** | **2.9E-07** | **-0.097** | **5.4E-07** | **-0.175** | **9.0E-09** | -0.046 | 3.2E-03 | -0.00048 | 1.3E-04 |
| Qigong | 21.4 | 9.4 | -0.018 | 2.3E-02 | -0.021 | 2.8E-03 | -0.011 | 3.0E-01 | -0.015 | 2.7E-01 | 0.00001 | 9.3E-01 |
| Others | 18.3 | 11.9 | 0.005 | 6.1E-01 | 0.023 | 1.5E-01 | -0.002 | 9.4E-01 | -0.012 | 3.9E-01 | -0.00002 | 8.9E-01 |
| Weight training | 15.4 | 9.1 | 0.000 | 9.8E-01 | -0.010 | 3.9E-01 | -0.007 | 7.6E-01 | 0.003 | 8.2E-01 | -0.00022 | 3.2E-01 |
| Badminton | 11.4 | 6.9 | 0.011 | 4.6E-01 | 0.027 | 3.0E-01 | 0.007 | 8.1E-01 | 0.031 | 1.3E-01 | -0.00049 | 5.9E-02 |
| Table tennis | 15.7 | 8.4 | -0.036 | 8.6E-03 | -0.038 | 4.7E-03 | -0.060 | 6.2E-03 | -0.040 | 7.7E-03 | **-0.00101** | **9.6E-06** |
| Basketball | 10.9 | 7.6 | -0.009 | 5.6E-01 | -0.041 | 3.2E-01 | -0.092 | 1.3E-02 | -0.025 | 5.7E-01 | -0.00064 | 1.9E-03 |
| Tennis | 16.7 | 8.4 | -0.004 | 8.0E-01 | -0.019 | 5.2E-01 | -0.061 | 2.0E-01 | -0.002 | 9.5E-01 | -0.00080 | 4.4E-03 |

**S9 Table.** Main associations of exercise frequencies per month with obesity measures (significant results with *p* < 9.1x10^-5^ are highlighted)

1. Having 1 more jog per month was associated with a 0.016 kg/m^2^ decrease in BMI. The regression model was built as BMI = $\beta_{0}$+$\beta_{GRS}$BMIGRS + $\beta_{E}$Jogging frequency + $\beta_{Int}$BMIGRS x Jogging frequency + $\boldsymbol{\beta}_{C}$**Covariates** + $\varepsilon$. Covariates adjusted in the regression model included sex, age, educational attainment, drinking status, smoking status, the first 10 PCs, 17 covariates regarding the frequencies per month of the other 17 kinds of exercise, and the interaction terms between BMIGRS and the frequencies of the 17 kinds of exercise. The jogging frequencies of subjects who did not choose jogging as their regular exercise were coded as 0.
