# Supplementary material for: Performing different kinds of physical exercise differentially attenuates the genetic effects on obesity measures: Evidence from 18,424 Taiwan Biobank participants
Source: PLoS Genet. 2019 Aug 1;15(8):e1008277. doi: 10.1371/journal.pgen.1008277 (PMC6675047; doi:10.1371/journal.pgen.1008277)
Supplement: S10 Table — (DOCX) [file pgen.1008277.s014.docx]

|  | Duration for each exercise (hours) | | BMI (kg/m^2^) | | Body fat % | | Waist circumference (cm) | | Hip circumference (cm) | | Waist-to-hip ratio | |
| --- | --- | --- | --- | --- | --- | --- | --- | --- | --- | --- | --- | --- |
|  | **Mean** | **Standard deviation** | ${\hat{\boldsymbol{\beta}}}_{\boldsymbol{E}}$ | ***P*-value** | ${\hat{\boldsymbol{\beta}}}_{\boldsymbol{E}}$ | ***P*-value** | ${\hat{\boldsymbol{\beta}}}_{\boldsymbol{E}}$ | ***P*-value** | ${\hat{\boldsymbol{\beta}}}_{\boldsymbol{E}}$ | ***P*-value** | ${\hat{\boldsymbol{\beta}}}_{\boldsymbol{E}}$ | ***P*-value** |
| Walking | 0.78 | 0.39 | 0.04 | 3.8E-01 | 0.11 | 1.6E-01 | 0.12 | 4.4E-01 | 0.06 | 5.6E-01 | 0.00179 | 1.4E-01 |
| Exercise walking | 0.81 | 0.38 | 0.05 | 5.2E-01 | -0.02 | 8.8E-01 | -0.29 | 2.6E-01 | 0.02 | 9.2E-01 | -0.00116 | 4.8E-01 |
| Jogging | 0.70 | 0.33 | -0.19 | 4.6E-02 | **-0.78** | **2.2E-06** | **-0.84** | **2.9E-05** | -0.65 | 2.6E-03 | **-0.00703** | **1.1E-06** |
| Cycling | 1.16 | 0.89 | -0.01 | 7.4E-01 | 0.02 | 8.5E-01 | -0.22 | 1.8E-01 | 0.07 | 6.5E-01 | -0.00179 | 1.1E-01 |
| Mountain climbing | 1.99 | 1.22 | -0.05 | 2.4E-01 | -0.13 | 1.3E-02 | -0.14 | 8.7E-02 | -0.08 | 2.0E-01 | -0.00100 | 5.9E-02 |
| Stretching exercise | 0.73 | 0.36 | -0.19 | 1.8E-01 | -0.41 | 1.8E-01 | -0.22 | 5.5E-01 | -0.10 | 7.2E-01 | 0.00200 | 4.3E-01 |
| International standard dancing | 1.28 | 0.61 | -0.04 | 5.9E-01 | -0.12 | 2.7E-01 | -0.31 | 3.9E-02 | 0.03 | 7.7E-01 | -0.00137 | 1.6E-01 |
| Swimming | 0.84 | 0.50 | 0.04 | 7.9E-01 | -0.30 | 2.2E-01 | -0.35 | 1.7E-01 | -0.09 | 6.0E-01 | -0.00326 | 2.1E-01 |
| Tai Chi | 1.13 | 0.54 | -0.21 | 4.2E-02 | -0.66 | 6.2E-04 | **-1.22** | **8.0E-06** | -0.71 | 3.5E-04 | -0.00473 | 1.8E-02 |
| Dance dance revolution | 1.01 | 0.43 | -0.07 | 4.8E-01 | -0.22 | 3.2E-01 | -0.51 | 6.9E-02 | -0.21 | 2.4E-01 | -0.00051 | 7.1E-01 |
| Yoga | 1.17 | 0.49 | **-0.67 ^1^** | **6.6E-07** | **-1.06** | **9.9E-07** | **-1.82** | **2.2E-07** | **-1.17** | **8.3E-06** | -0.00440 | 5.8E-04 |
| Qigong | 1.01 | 0.46 | -0.29 | 6.4E-02 | -0.70 | 6.6E-03 | -0.47 | 2.0E-01 | -0.26 | 3.6E-01 | 0.00079 | 5.8E-01 |
| Others | 0.96 | 0.65 | 0.10 | 5.8E-01 | 0.25 | 3.7E-01 | 0.15 | 7.3E-01 | -0.09 | 6.6E-01 | 0.00193 | 4.7E-01 |
| Weight training | 0.80 | 0.48 | -0.01 | 9.2E-01 | -0.33 | 1.3E-01 | -0.16 | 6.9E-01 | 0.14 | 6.0E-01 | -0.00772 | 3.3E-02 |
| Badminton | 1.40 | 0.60 | 0.20 | 2.2E-01 | 0.13 | 5.7E-01 | 0.03 | 9.2E-01 | 0.67 | 3.2E-02 | -0.00562 | 3.5E-02 |
| Table tennis | 1.34 | 0.59 | -0.32 | 2.8E-02 | -0.42 | 2.4E-02 | -0.76 | 8.9E-03 | -0.61 | 9.4E-03 | -0.00329 | 1.2E-01 |
| Basketball | 1.40 | 0.68 | 0.04 | 7.7E-01 | 0.17 | 6.4E-01 | -0.15 | 6.4E-01 | 0.69 | 7.1E-02 | -0.00407 | 2.3E-01 |
| Tennis | 1.41 | 0.64 | 0.17 | 4.3E-01 | 0.11 | 7.5E-01 | -0.34 | 5.1E-01 | 0.32 | 4.5E-01 | -0.00580 | 9.6E-02 |

**S10 Table.** Main associations of the exercise duration (in hours) with obesity measures (significant results with *p* < 9.1x10^-5^ are highlighted)

1. Performing 1 more hour in each yoga practice was associated with a 0.67 kg/m^2^ decrease in BMI. The regression model was built as BMI = $\beta_{0}$+$\beta_{GRS}$BMIGRS + $\beta_{E}$Yoga duration + $\beta_{Int}$BMIGRS x Yoga duration + $\boldsymbol{\beta}_{C}$**Covariates** + $\varepsilon$. Covariates adjusted in the regression model included sex, age, educational attainment, drinking status, smoking status, the first 10 PCs, 17 covariates regarding the duration (in hours) of the other 17 kinds of exercise, and the interaction terms between BMIGRS and the duration of the 17 kinds of exercise. The yoga duration of subjects who did not engage in yoga was coded as 0.
