# Supplementary material for: Performing different kinds of physical exercise differentially attenuates the genetic effects on obesity measures: Evidence from 18,424 Taiwan Biobank participants
Source: PLoS Genet. 2019 Aug 1;15(8):e1008277. doi: 10.1371/journal.pgen.1008277 (PMC6675047; doi:10.1371/journal.pgen.1008277)
Supplement: S11 Table — (DOCX) [file pgen.1008277.s015.docx]

|  | | |  | BMI (kg/m^2^) | | Body fat % | | Waist circumference (cm) | | Hip circumference (cm) | | Waist-to-hip ratio | |
| --- | --- | --- | --- | --- | --- | --- | --- | --- | --- | --- | --- | --- | --- |
|  | |  | **No. of subjects** | ${\hat{\boldsymbol{\beta}}}_{\boldsymbol{Int}}$ | **GRS-M *P*-value** | ${\hat{\boldsymbol{\beta}}}_{\boldsymbol{Int}}$ | **GRS-M *P*-value** | ${\hat{\boldsymbol{\beta}}}_{\boldsymbol{Int}}$ | **GRS-M *P*-value** | ${\hat{\boldsymbol{\beta}}}_{\boldsymbol{Int}}$ | **GRS-M *P*-value** | ${\hat{\boldsymbol{\beta}}}_{\boldsymbol{Int}}$ | **GRS-M *P*-value** |
| Regular exercise | | Men | 3,895 | -0.42 | 5.0E-17 | -0.62 | 1.1E-9 | -0.79 | 2.1E-09 | -0.68 | 5.4E-10 | -0.0015 | 0.16 |
|  |  | Women | 3,757 | -0.43 | 3.3E-18 | -0.60 | 3.1E-8 | -0.60 | 2.3E-06 | -0.69 | 1.8E-10 | 0.0004 | 0.75 |
|  | Specific analysis for kinds of exercise: Some subjects engage in 2 or 3 kinds of regular exercise.  The following 18 kinds of exercise were sorted according to popularity. | | | | | | | | | | | | |
| Walking | | Men | 1,247 | -0.25 | 9.7E-05 | -0.23 | 4.4E-02 | -0.62 | 1.3E-03 | -0.35 | 6.0E-03 | 0.0006 | 0.68 |
|  |  | Women | 1,390 | -0.24 | 1.2E-04 | -0.13 | 1.9E-01 | -0.45 | 1.2E-02 | -0.26 | 2.1E-02 | 0.0041 | 0.01 |
| Exercise walking | | Men | 753 | -0.51 | 1.7E-07 | -0.73 | 4.9E-05 | -1.06 | 1.0E-03 | -0.80 | 8.8E-05 | -0.0057 | 0.00 |
|  |  | Women | 686 | -0.21 | 3.7E-02 | -0.45 | 1.7E-02 | -0.74 | 2.3E-02 | -0.44 | 4.3E-02 | -0.0001 | 0.98 |
| Jogging | | Men | 898 | -0.39 | 1.7E-06 | -0.26 | 7.8E-02 | -0.64 | 6.2E-04 | -0.64 | 7.4E-04 | -0.0024 | 0.07 |
|  |  | Women | 209 | -0.40 | 2.1E-02 | -0.93 | 2.8E-03 | -0.43 | 2.4E-01 | -1.30 | 5.8E-04 | 0.0000 | 0.99 |
| Cycling | | Men | 678 | -0.27 | 6.2E-02 | -0.39 | 4.6E-02 | -0.56 | 3.6E-02 | -0.25 | 3.1E-01 | -0.0034 | 0.11 |
|  |  | Women | 311 | -0.20 | 3.4E-01 | -0.39 | 1.9E-01 | -0.10 | 8.0E-01 | -0.12 | 7.7E-01 | -0.0035 | 0.28 |
| Mountain climbing | | Men | 360 | -0.46 | 5.0E-04 | -0.56 | 1.5E-03 | -0.57 | 4.0E-02 | -0.35 | 8.5E-02 | -0.0022 | 0.38 |
|  |  | Women | 268 | -0.71 | 2.5E-05 | -0.24 | 2.7E-01 | -1.07 | 1.3E-03 | -0.89 | 2.5E-04 | -0.0038 | 0.21 |
| Stretching exercise | | Men | 204 | -0.39 | 5.6E-02 | -0.66 | 7.9E-02 | -0.59 | 2.5E-01 | -1.10 | 4.7E-03 | -0.0034 | 0.29 |
|  |  | Women | 398 | -0.25 | 7.5E-02 | -0.50 | 1.2E-01 | -0.67 | 7.4E-02 | -0.01 | 9.9E-01 | -0.0052 | 0.03 |
| International standard dancing | | Men | 71 | -0.47 | 7.1E-02 | -0.60 | 1.8E-01 | 0.12 | 8.6E-01 | -0.05 | 9.1E-01 | -0.0068 | 0.11 |
|  |  | Women | 442 | -0.46 | 2.1E-06 | -0.74 | 5.1E-06 | -0.65 | 5.2E-03 | -0.48 | 4.1E-03 | -0.0033 | 0.02 |
| Swimming | | Men | 323 | -0.11 | 5.4E-01 | -0.41 | 1.6E-01 | 0.93 | 3.7E-03 | -0.18 | 4.3E-01 | 0.0100 | 0.00 |
|  |  | Women | 163 | -0.71 | 1.1E-02 | -0.58 | 2.0E-01 | 0.00 | 1.0E+00 | -0.25 | 4.1E-01 | 0.0001 | 0.97 |
| Tai Chi | | Men | 250 | -0.52 | 9.8E-03 | -0.74 | 2.8E-02 | -0.50 | 3.1E-01 | -0.48 | 1.8E-01 | -0.0063 | 0.04 |
|  |  | Women | 199 | -0.68 | 1.2E-03 | -1.37 | 4.0E-04 | -1.51 | 6.5E-03 | -1.47 | 1.1E-04 | -0.0057 | 0.18 |
| Dance dance revolution | | Men | 35 | 0.14 | 6.8E-01 | -0.72 | 4.0E-01 | -0.80 | 5.2E-01 | -0.16 | 8.0E-01 | -0.0121 | 0.06 |
|  |  | Women | 385 | -0.38 | 1.8E-03 | -0.83 | 5.1E-03 | -0.82 | 1.9E-02 | -0.75 | 6.0E-04 | 0.0021 | 0.19 |
| Yoga | | Men | 39 | -0.57 | 4.3E-01 | 0.87 | 1.0E-01 | -0.72 | 6.0E-01 | -1.39 | 1.8E-01 | 0.0004 | 0.94 |
|  |  | Women | 340 | -0.76 | 6.9E-05 | -0.05 | 8.1E-01 | -1.41 | 2.2E-03 | -0.72 | 6.2E-02 | 0.0007 | 0.71 |
| Qigong | | Men | 137 | -0.14 | 6.5E-01 | -0.25 | 3.5E-01 | -0.64 | 3.6E-01 | -0.79 | 1.3E-01 | -0.0016 | 0.72 |
|  |  | Women | 240 | -0.50 | 2.0E-02 | -0.35 | 7.1E-02 | -0.77 | 1.4E-01 | -1.41 | 3.6E-03 | -0.0045 | 0.15 |
| Others | | Men | 118 | -0.32 | 1.9E-01 | -1.04 | 2.5E-02 | 0.05 | 9.5E-01 | 0.30 | 5.4E-01 | -0.0075 | 0.07 |
|  |  | Women | 167 | -0.22 | 2.8E-01 | -0.53 | 1.8E-01 | -1.49 | 3.9E-02 | 0.85 | 5.3E-02 | -0.0035 | 0.45 |
| Weight training | | Men | 159 | -0.36 | 2.1E-02 | -0.48 | 7.0E-02 | -1.12 | 1.2E-02 | -0.45 | 1.5E-01 | 0.0002 | 0.96 |
|  |  | Women | 59 | -0.26 | 2.7E-01 | -0.71 | 8.4E-02 | 0.05 | 9.4E-01 | -0.47 | 3.1E-01 | 0.0130 | 0.15 |
| Badminton | | Men | 161 | -0.38 | 1.6E-01 | -0.43 | 2.4E-01 | -0.38 | 3.7E-01 | -0.66 | 2.0E-01 | 0.0082 | 0.07 |
|  |  | Women | 43 | -0.07 | 8.9E-01 | -0.37 | 5.8E-01 | -0.34 | 7.4E-01 | -0.29 | 7.8E-01 | 0.0005 | 0.96 |
| Table tennis | | Men | 129 | -0.54 | 3.6E-02 | -0.48 | 1.7E-01 | -0.63 | 1.9E-01 | -0.77 | 2.6E-02 | 0.0106 | 0.01 |
|  |  | Women | 40 | -0.87 | 5.3E-02 | -0.85 | 1.7E-01 | -1.35 | 2.8E-01 | -0.37 | 5.3E-01 | 0.0012 | 0.91 |
| Basketball | | Men | 116 | 0.40 | 1.1E-01 | -0.55 | 2.6E-01 | 1.12 | 5.8E-02 | -1.06 | 6.5E-02 | -0.0050 | 0.11 |
|  |  | Women | 3 | 0.59 | 5.7E-01 | -3.68 | 2.8E-01 | 3.10 | 2.0E-01 | -6.02 | 7.2E-02 | -0.0026 | 0.85 |
| Tennis | | Men | 89 | -0.51 | 1.1E-01 | -1.43 | 1.8E-02 | 2.54 | 3.0E-02 | 0.99 | 1.4E-01 | -0.0023 | 0.43 |
|  |  | Women | 21 | 0.15 | 8.0E-01 | -0.68 | 5.8E-01 | -1.19 | 5.8E-01 | 1.31 | 3.9E-01 | -0.0048 | 0.38 |

**S11 Table.** Interaction between GRS and exercise on each obesity measure (stratified by sex)
