# Supplementary material for: Performing different kinds of physical exercise differentially attenuates the genetic effects on obesity measures: Evidence from 18,424 Taiwan Biobank participants
Source: PLoS Genet. 2019 Aug 1;15(8):e1008277. doi: 10.1371/journal.pgen.1008277 (PMC6675047; doi:10.1371/journal.pgen.1008277)
Supplement: S12 Table — (DOCX) [file pgen.1008277.s016.docx]

|  | |  | BMI (kg/m^2^) | | Body fat % | | Waist circumference (cm) | | Hip circumference (cm) | | Waist-to-hip ratio | |
| --- | --- | --- | --- | --- | --- | --- | --- | --- | --- | --- | --- | --- |
|  |  | **No. of subjects** | ${\hat{\boldsymbol{\beta}}}_{\boldsymbol{Int}}$ | **GRS-M *P*-value** | ${\hat{\boldsymbol{\beta}}}_{\boldsymbol{Int}}$ | **GRS-M *P*-value** | ${\hat{\boldsymbol{\beta}}}_{\boldsymbol{Int}}$ | **GRS-M *P*-value** | ${\hat{\boldsymbol{\beta}}}_{\boldsymbol{Int}}$ | **GRS-M *P*-value** | ${\hat{\boldsymbol{\beta}}}_{\boldsymbol{Int}}$ | **GRS-M *P*-value** |
| Walking | Men | 1,247 | -0.012 | 4.7E-05 | -0.010 | 3.2E-02 | -0.026 | 1.4E-03 | -0.015 | 7.6E-03 | 0.00001 | 8.55E-01 |
|  | Women | 1,390 | -0.011 | 6.0E-05 | -0.008 | 6.3E-02 | -0.017 | 3.1E-02 | -0.015 | 5.3E-03 | 0.00011 | 1.03E-01 |
| Exercise walking | Men | 753 | -0.024 | 2.2E-05 | -0.036 | 3.2E-05 | -0.055 | 1.8E-04 | -0.036 | 1.2E-04 | -0.00020 | 6.48E-03 |
|  | Women | 686 | -0.017 | 8.8E-03 | -0.022 | 2.4E-02 | -0.033 | 3.7E-02 | -0.026 | 1.8E-02 | -0.00006 | 4.78E-01 |
| Jogging | Men | 898 | -0.022 | 6.9E-06 | -0.007 | 4.0E-01 | -0.031 | 4.4E-02 | -0.034 | 1.8E-03 | -0.00002 | 8.08E-01 |
|  | Women | 209 | -0.028 | 4.5E-03 | -0.057 | 1.9E-03 | -0.064 | 4.4E-02 | -0.095 | 3.9E-05 | -0.00010 | 6.12E-01 |
| Cycling | Men | 678 | -0.019 | 7.4E-03 | -0.019 | 5.7E-02 | -0.036 | 7.5E-02 | -0.007 | 5.2E-01 | -0.00028 | 1.78E-02 |
|  | Women | 311 | -0.010 | 4.0E-01 | -0.014 | 3.9E-01 | -0.020 | 4.9E-01 | -0.017 | 3.0E-01 | -0.00008 | 6.93E-01 |
| Mountain climbing | Men | 360 | -0.029 | 3.8E-03 | -0.029 | 3.2E-02 | -0.041 | 6.6E-02 | -0.010 | 5.2E-01 | -0.00020 | 2.58E-01 |
|  | Women | 268 | -0.061 | 3.7E-04 | -0.025 | 1.6E-01 | -0.054 | 1.4E-01 | -0.062 | 1.9E-03 | -0.00013 | 5.85E-01 |
| Stretching exercise | Men | 204 | -0.018 | 6.4E-02 | -0.032 | 4.1E-02 | -0.029 | 1.3E-01 | -0.022 | 2.3E-01 | -0.00016 | 2.16E-01 |
|  | Women | 398 | -0.014 | 6.1E-02 | -0.024 | 1.0E-01 | -0.012 | 4.1E-01 | -0.011 | 4.9E-01 | -0.00023 | 3.21E-02 |
| International standard dancing | Men | 71 | -0.045 | 1.6E-02 | -0.069 | 2.0E-02 | 0.001 | 9.8E-01 | -0.016 | 5.9E-01 | -0.00022 | 5.54E-01 |
|  | Women | 442 | -0.022 | 2.6E-05 | -0.033 | 1.5E-04 | -0.037 | 3.4E-03 | -0.032 | 5.9E-04 | 0.00003 | 8.38E-01 |
| Swimming | Men | 323 | -0.010 | 3.3E-01 | -0.028 | 9.2E-02 | 0.029 | 7.5E-02 | -0.023 | 2.7E-01 | 0.00057 | 1.03E-04 |
|  | Women | 163 | -0.042 | 5.8E-03 | -0.027 | 3.0E-01 | 0.030 | 2.5E-01 | -0.028 | 3.8E-01 | -0.00002 | 9.29E-01 |
| Tai Chi | Men | 250 | -0.019 | 4.4E-02 | -0.034 | 2.9E-02 | -0.019 | 3.9E-01 | -0.016 | 3.0E-01 | -0.00025 | 8.03E-02 |
|  | Women | 199 | -0.038 | 2.3E-03 | -0.065 | 3.0E-03 | -0.063 | 2.6E-02 | -0.078 | 6.9E-05 | -0.00025 | 2.36E-01 |
| Dance dance revolution | Men | 35 | 0.020 | 5.7E-01 | -0.095 | 3.0E-01 | -0.096 | 3.2E-01 | -0.023 | 6.7E-01 | -0.00060 | 2.00E-01 |
|  | Women | 385 | -0.023 | 1.3E-03 | -0.054 | 3.9E-03 | -0.042 | 5.0E-02 | -0.036 | 5.1E-03 | 0.00014 | 1.72E-01 |
| Yoga | Men | 39 | -0.042 | 3.1E-01 | -0.015 | 7.3E-01 | -0.037 | 6.6E-01 | -0.033 | 3.8E-01 | -0.00014 | 7.20E-01 |
|  | Women | 340 | -0.062 | 1.1E-05 | -0.038 | 5.2E-02 | -0.137 | 1.2E-04 | -0.047 | 5.8E-03 | 0.00006 | 6.92E-01 |
| Qigong | Men | 137 | 0.004 | 7.7E-01 | -0.014 | 1.7E-01 | 0.002 | 9.2E-01 | -0.031 | 1.4E-01 | 0.00005 | 7.90E-01 |
|  | Women | 240 | -0.018 | 5.3E-02 | -0.010 | 2.5E-01 | 0.015 | 2.7E-01 | -0.048 | 1.9E-02 | -0.00023 | 9.88E-02 |
| Others | Men | 118 | -0.027 | 4.8E-02 | 0.033 | 5.9E-02 | -0.019 | 5.5E-01 | 0.011 | 5.5E-01 | -0.00041 | 2.23E-02 |
|  | Women | 167 | -0.010 | 4.7E-01 | 0.018 | 4.8E-01 | -0.081 | 2.6E-02 | 0.020 | 3.9E-01 | 0.00002 | 9.30E-01 |
| Weight training | Men | 159 | -0.016 | 5.5E-02 | -0.023 | 9.8E-02 | -0.055 | 1.2E-02 | -0.024 | 1.3E-01 | 0.00015 | 5.55E-01 |
|  | Women | 59 | -0.020 | 1.5E-01 | -0.048 | 2.9E-02 | -0.025 | 5.8E-01 | -0.045 | 1.3E-01 | 0.00066 | 2.71E-01 |
| Badminton | Men | 161 | -0.021 | 2.1E-01 | -0.085 | 4.7E-03 | -0.051 | 1.3E-01 | -0.044 | 6.1E-02 | -0.00002 | 9.36E-01 |
|  | Women | 43 | -0.053 | 1.0E-01 | 0.012 | 8.4E-01 | -0.065 | 4.2E-01 | 0.008 | 9.0E-01 | -0.00045 | 4.96E-01 |
| Table tennis | Men | 129 | -0.030 | 5.5E-02 | -0.022 | 1.9E-01 | -0.024 | 2.9E-01 | -0.045 | 2.2E-02 | 0.00079 | 6.44E-04 |
|  | Women | 40 | -0.046 | 1.3E-01 | -0.051 | 8.7E-02 | -0.037 | 6.2E-01 | -0.023 | 5.3E-01 | -0.00034 | 5.61E-01 |
| Basketball | Men | 116 | 0.023 | 2.2E-01 | -0.038 | 3.2E-01 | 0.057 | 1.9E-01 | -0.099 | 3.3E-02 | -0.00042 | 5.08E-02 |
|  | Women | 3 | 0.121 | 5.7E-01 | -0.233 | 4.2E-01 | 0.382 | 3.9E-01 | 0.616 | 2.4E-01 | -0.00095 | 6.29E-01 |
| Tennis | Men | 89 | -0.040 | 1.9E-02 | -0.077 | 1.4E-02 | 0.117 | 5.8E-02 | -0.017 | 5.5E-01 | 0.00052 | 4.38E-02 |
|  | Women | 21 | -0.004 | 9.1E-01 | -0.067 | 4.3E-01 | -0.051 | 6.3E-01 | 0.021 | 7.3E-01 | -0.00004 | 9.40E-01 |

**S12 Table.** Interaction between GRS and exercise frequency per month (stratified by sex)
