# Supplementary material for: Performing different kinds of physical exercise differentially attenuates the genetic effects on obesity measures: Evidence from 18,424 Taiwan Biobank participants
Source: PLoS Genet. 2019 Aug 1;15(8):e1008277. doi: 10.1371/journal.pgen.1008277 (PMC6675047; doi:10.1371/journal.pgen.1008277)
Supplement: S13 Table — (DOCX) [file pgen.1008277.s017.docx]

|  | |  | BMI (kg/m^2^) | | Body fat % | | Waist circumference (cm) | | Hip circumference (cm) | | Waist-to-hip ratio | |
| --- | --- | --- | --- | --- | --- | --- | --- | --- | --- | --- | --- | --- |
|  |  | **No. of subjects** | ${\hat{\boldsymbol{\beta}}}_{\boldsymbol{Int}}$ | **GRS-M *P*-value** | ${\hat{\boldsymbol{\beta}}}_{\boldsymbol{Int}}$ | **GRS-M *P*-value** | ${\hat{\boldsymbol{\beta}}}_{\boldsymbol{Int}}$ | **GRS-M *P*-value** | ${\hat{\boldsymbol{\beta}}}_{\boldsymbol{Int}}$ | **GRS-M *P*-value** | ${\hat{\boldsymbol{\beta}}}_{\boldsymbol{Int}}$ | **GRS-M *P*-value** |
| Walking | Men | 1,247 | -0.24 | 5.1E-04 | -0.10 | 3.3E-01 | -0.68 | 1.3E-03 | -0.33 | 1.2E-02 | 0.00074 | 6.4E-01 |
|  | Women | 1,390 | -0.26 | 5.1E-04 | -0.16 | 1.1E-01 | -0.51 | 1.5E-02 | -0.33 | 1.7E-02 | 0.00622 | 3.1E-04 |
| Exercise walking | Men | 753 | -0.50 | 3.4E-06 | -0.69 | 5.4E-04 | -0.98 | 3.3E-03 | -0.83 | 2.6E-04 | -0.00516 | 1.2E-02 |
|  | Women | 686 | -0.23 | 2.9E-02 | -0.57 | 9.4E-03 | -0.66 | 5.7E-02 | -0.57 | 9.3E-03 | -0.00084 | 7.1E-01 |
| Jogging | Men | 898 | -0.55 | 1.2E-06 | -0.45 | 1.0E-02 | -0.75 | 8.8E-04 | -0.74 | 1.5E-03 | -0.00299 | 6.0E-02 |
|  | Women | 209 | -0.76 | 2.5E-03 | -1.51 | 8.5E-04 | -0.44 | 3.6E-01 | -1.63 | 1.4E-03 | 0.00101 | 7.4E-01 |
| Cycling | Men | 678 | -0.05 | 3.0E-01 | -0.21 | 9.8E-02 | -0.33 | 4.5E-02 | -0.20 | 2.1E-01 | -0.00202 | 8.5E-02 |
|  | Women | 311 | -0.13 | 2.6E-01 | -0.02 | 9.5E-01 | -0.24 | 5.5E-01 | -0.33 | 4.2E-01 | 0.00292 | 3.2E-01 |
| Mountain climbing | Men | 360 | -0.23 | 1.4E-04 | -0.27 | 8.5E-04 | -0.36 | 3.2E-03 | -0.12 | 1.4E-01 | -0.00170 | 3.4E-02 |
|  | Women | 268 | -0.25 | 7.7E-04 | -0.07 | 4.4E-01 | -0.46 | 1.9E-03 | -0.42 | 4.4E-04 | -0.00144 | 8.7E-02 |
| Stretching exercise | Men | 204 | -0.54 | 6.0E-02 | -0.39 | 4.0E-01 | -1.06 | 1.2E-01 | -1.48 | 3.4E-03 | -0.00487 | 2.4E-01 |
|  | Women | 398 | -0.25 | 1.3E-01 | -0.59 | 1.4E-01 | -0.77 | 7.3E-02 | 0.04 | 9.0E-01 | -0.00540 | 4.7E-02 |
| International standard dancing | Men | 71 | -0.25 | 8.8E-02 | -0.38 | 8.5E-02 | 0.15 | 6.8E-01 | 0.02 | 9.4E-01 | -0.00321 | 1.8E-01 |
|  | Women | 442 | -0.31 | 6.6E-06 | -0.49 | 1.9E-05 | -0.55 | 2.2E-03 | -0.33 | 4.8E-03 | -0.00242 | 2.0E-02 |
| Swimming | Men | 323 | 0.00 | 9.8E-01 | -0.22 | 3.8E-01 | 0.70 | 1.5E-02 | -0.27 | 2.2E-01 | 0.00872 | 8.1E-04 |
|  | Women | 163 | -1.12 | 3.1E-03 | -0.24 | 6.6E-01 | -0.31 | 6.3E-01 | -0.43 | 2.4E-01 | -0.00091 | 8.7E-01 |
| Tai Chi | Men | 250 | -0.26 | 9.3E-02 | -0.70 | 7.5E-03 | -0.69 | 7.6E-02 | -0.50 | 6.5E-02 | -0.00396 | 1.2E-01 |
|  | Women | 199 | -0.44 | 8.7E-04 | -0.81 | 7.7E-03 | -1.18 | 8.6E-03 | -1.12 | 4.0E-04 | -0.00255 | 4.6E-01 |
| Dance dance revolution | Men | 35 | -0.04 | 9.0E-01 | -0.86 | 2.7E-01 | -0.83 | 4.4E-01 | 0.00 | 9.9E-01 | -0.01339 | 4.9E-02 |
|  | Women | 385 | -0.25 | 2.2E-02 | -0.55 | 3.3E-02 | -0.69 | 2.8E-02 | -0.58 | 3.0E-03 | 0.00175 | 2.4E-01 |
| Yoga | Men | 39 | -0.71 | 2.2E-01 | 0.22 | 7.4E-01 | -0.40 | 7.5E-01 | -1.32 | 5.8E-02 | 0.00230 | 5.7E-01 |
|  | Women | 340 | -0.65 | 2.7E-05 | -0.51 | 3.0E-02 | -1.10 | 2.0E-03 | -0.60 | 5.2E-02 | 0.00059 | 6.5E-01 |
| Qigong | Men | 137 | -0.18 | 3.7E-01 | -0.37 | 2.6E-01 | -0.46 | 4.0E-01 | -0.79 | 9.3E-02 | -0.00250 | 3.3E-01 |
|  | Women | 240 | -0.45 | 2.3E-02 | -0.68 | 2.7E-02 | -0.95 | 6.5E-02 | -1.25 | 8.0E-03 | -0.00277 | 1.1E-01 |
| Others | Men | 118 | -0.30 | 3.7E-01 | -0.82 | 7.5E-02 | -0.31 | 6.4E-01 | 0.17 | 6.8E-01 | -0.00381 | 3.0E-01 |
|  | Women | 167 | -0.30 | 2.5E-01 | -0.59 | 1.1E-01 | -1.55 | 9.8E-03 | 0.29 | 2.6E-01 | -0.00654 | 9.0E-02 |
| Weight training | Men | 159 | -0.35 | 1.9E-02 | -0.32 | 2.0E-01 | -1.45 | 2.6E-03 | -0.50 | 6.1E-02 | -0.00305 | 4.4E-01 |
|  | Women | 59 | 0.07 | 8.3E-01 | -0.26 | 6.6E-01 | 0.01 | 9.9E-01 | -0.15 | 8.1E-01 | -0.00699 | 3.6E-01 |
| Badminton | Men | 161 | -0.26 | 1.6E-01 | -0.43 | 8.2E-02 | -0.18 | 4.8E-01 | -0.36 | 2.5E-01 | 0.00516 | 5.4E-02 |
|  | Women | 43 | -0.19 | 5.6E-01 | -0.13 | 8.4E-01 | -0.33 | 7.1E-01 | 0.08 | 9.4E-01 | 0.00589 | 5.0E-01 |
| Table tennis | Men | 129 | -0.31 | 7.8E-02 | -0.26 | 2.7E-01 | -0.38 | 2.8E-01 | -0.64 | 2.3E-02 | 0.00520 | 3.7E-02 |
|  | Women | 40 | -0.68 | 4.8E-02 | -0.65 | 8.9E-02 | -0.64 | 4.1E-01 | -0.40 | 3.6E-01 | 0.00529 | 3.9E-01 |
| Basketball | Men | 116 | 0.32 | 3.9E-02 | -0.50 | 1.1E-01 | 0.73 | 4.0E-02 | -0.91 | 2.4E-02 | -0.00516 | 1.5E-01 |
|  | Women | 3 | 0.85 | 6.7E-01 | -5.07 | 3.6E-01 | 4.84 | 3.0E-01 | 6.53 | 2.5E-01 | 0.34970 | 1.5E-01 |
| Tennis | Men | 89 | -0.52 | 2.2E-02 | -0.92 | 1.1E-02 | 0.52 | 3.9E-01 | 0.56 | 1.9E-01 | -0.00132 | 6.9E-01 |
|  | Women | 21 | 0.08 | 8.6E-01 | -0.96 | 3.3E-01 | 1.30 | 2.8E-01 | 1.44 | 2.9E-01 | 0.00279 | 7.2E-01 |

**S13 Table.** Interaction between GRS and exercise duration (in hours) (stratified by sex)
